# Iron, copper, zinc, and manganese transport and regulation in pathogenic Enterobacteria: correlations between strains, site of infection and the relative importance of the different metal transport systems for virulence

**DOI:** 10.3389/fcimb.2013.00090

**Published:** 2013-12-05

**Authors:** Gaëlle Porcheron, Amélie Garénaux, Julie Proulx, Mourad Sabri, Charles M. Dozois

**Affiliations:** ^1^INRS-Institut Armand FrappierLaval, QC, Canada; ^2^Centre de Recherche en Infectiologie Porcine et Aviaire, Faculté de Médecine Vétérinaire, Université de MontréalSaint-Hyacinthe, QC, Canada; ^3^Groupe de Recherche sur les Maladies Infectieuses du Porc, Faculté de Médecine Vétérinaire, Université de MontréalSaint-Hyacinthe, QC, Canada

**Keywords:** metal transporters, iron, copper, zinc, manganese, Enterobacteria, regulation, virulence

## Abstract

For all microorganisms, acquisition of metal ions is essential for survival in the environment or in their infected host. Metal ions are required in many biological processes as components of metalloproteins and serve as cofactors or structural elements for enzymes. However, it is critical for bacteria to ensure that metal uptake and availability is in accordance with physiological needs, as an imbalance in bacterial metal homeostasis is deleterious. Indeed, host defense strategies against infection either consist of metal starvation by sequestration or toxicity by the highly concentrated release of metals. To overcome these host strategies, bacteria employ a variety of metal uptake and export systems and finely regulate metal homeostasis by numerous transcriptional regulators, allowing them to adapt to changing environmental conditions. As a consequence, iron, zinc, manganese, and copper uptake systems significantly contribute to the virulence of many pathogenic bacteria. However, during the course of our experiments on the role of iron and manganese transporters in extraintestinal *Escherichia coli* (ExPEC) virulence, we observed that depending on the strain tested, the importance of tested systems in virulence may be different. This could be due to the different set of systems present in these strains, but literature also suggests that as each pathogen must adapt to the particular microenvironment of its site of infection, the role of each acquisition system in virulence can differ from a particular strain to another. In this review, we present the systems involved in metal transport by Enterobacteria and the main regulators responsible for their controlled expression. We also discuss the relative role of these systems depending on the pathogen and the tissues they infect.

## Introduction

Metal ions such as iron, copper, zinc, and manganese, are involved in many crucial biological processes and are necessary for the survival of all living organisms. They are ubiquitously found in all organisms, nearly exclusively as constituents of proteins, including enzymes, storage proteins and transcription factors (Hood and Skaar, 2012). Due to the unique redox potential of some of these transition metals, many serve important roles as cofactors in enzymes and it is estimated that 30–45% of known enzymes are metalloproteins whose functions require a metal co-factor (Klein and Lewinson, 2011). However, transition metals are toxic at high intracellular concentrations, as they perturb the cellular redox potential and produce highly reactive hydroxyl radicals. Therefore, all organisms require mechanisms for sensing small fluctuations in metal levels to maintain a controlled balance of uptake, efflux, and sequestration and to ensure that metal availability is in accordance with physiological needs. This ability to sense metal ions is particularly important for bacterial pathogens to invade their hosts and cause disease. The ability of bacteria to colonize specific environments depends on their ability to obtain required nutrients. The strict requirement for these elements during pathogenesis is due to their involvement in processes ranging from bacterial metabolism to virulence factor expression (Waldron and Robinson, 2009; Kehl-Fie and Skaar, 2010). However, during infection, the host also produces proteins that are able to chelate metal ions and thus, can restrict the availability of essential metals from invading pathogens. Moreover, the toxicity of metals such as copper can be used as a host defense mechanism to promote bacterial killing. Nutrient limitation by the host and nutrient acquisition by pathogenic bacteria are therefore, crucial processes in the pathogenesis of bacterial infectious diseases. The host and the bacterial pathogen might thus, be envisioned as living through a constant competition for the essential metal nutrients. As a result of this competition, bacteria have developed sophisticated acquisition systems to scavenge essential metals from the environment. These include constitutively expressed or inducible low- and high-affinity transport systems for chelated or free metals. Moreover, efflux systems are used to eliminate the excess metal ions which might become toxic for the bacterial cell. Acquisition systems are up-regulated during metal starvation, and efflux pumps are activated when metals are in excess (Wakeman and Skaar, 2012).

The *Enterobacteriaceae* comprise a large family of Gram-negative bacteria that include pathogenic species such as pathogenic *Escherichia coli* and *Shigella* spp., *Salmonella enterica, Klebsiella pneumoniae*, and others. While *E. coli* is a member of the commensal intestinal flora, some *E. coli* strains have evolved pathogenic mechanisms to colonize humans and animals. *E. coli* strains can cause either intestinal infections (caused collectively by different types of Intestinal Pathogenic *E. coli* [IPEC]) or extraintestinal infections (caused by Extraintestinal Pathogenic *E. coli* [ExPEC]). Eight pathotypes of IPEC are currently described [see Clements et al. (2012) for review]. ExPEC strains contain 3 major pathotypes: UroPathogenic *E. coli* (UPEC), Neonatal Meningitis *E. coli* (NMEC), and Avian Pathogenic *E. coli* (APEC). These strains are responsible for urinary tract infections, meningitis in neonates and avian respiratory tract infections, respectively. ExPEC infections can also lead to septicaemia. ExPEC have an enhanced ability to cause infection outside of the intestinal tract and can infect the urinary tract, the bloodstream, and the cerebrospinal fluid of human and other animal hosts (Dho-Moulin and Fairbrother, 1999; Russo and Johnson, 2000). *Salmonella* is a major pathogen of both animals and humans, and is the cause of typhoid fever, paratyphoid fever, and the foodborne illness salmonellosis. *Salmonella* strains reach the gastrointestinal epithelium and trigger gastrointestinal diseases. They are able to invade the intestinal epithelium and to survive within phagocytes (Liu et al., 2013). *Shigella* species are responsible for bacillary dysentery. To infect their host, they have to be able to survive in the environment (such as contaminated water) as well as inside host epithelial cells (Payne et al., 2006). Seventeen different species of *Yersinia* have been reported, of which three have been shown to be pathogenic to humans and animals. These are *Y. enterocolitica* and *Y. pseudotuberculosis*, and the most virulent and invasive, *Y*. *pestis*. The latter causes highly fatal pneumonic, bubonic and septicemic plague, while the first two are responsible for a wide range of diseases ranging from mild diarrhea to enterocolitis (Mikula et al., 2012). *Klebsiella*, particularly *K. pneumoniae*, frequently cause human nosocomial infections. Nosocomial *Klebsiella* infections most commonly involve the urinary and respiratory tracts (Podschun and Ullmann, 1998). Members of the genus *Serratia*, particularly *Serratia marcesens*, cause important infections in humans, animals, and insects. *S. marcescens* is an opportunistic pathogen causing clinical diseases such as urinary tract infections and pneumonia (Mahlen, 2011). Several species of *Proteus* bacteria infect humans. The most frequently linked with human disease, *Proteus mirabilis*, is the causative agent of nosocomial and urinary tract infections (Jacobsen and Shirtliff, 2011). The genus *Cronobacter* is very diverse and comprises pathogens causing severe meningitis, septicemia, or necrotizing enterocolitis in neonates and infants (Grim et al., 2012). The *Enterobacteriaceae* family contains several other genera that are pathogenic, but they will not be described herein, as studies on metal transporters in these other genera are very limited.

This review will focus on the transport systems of four essential metals, i.e., iron, manganese, zinc, and copper, identified in pathogenic Enterobacteria, and the control between uptake and export of these metals which is needed to ensure physiological needs while countering metal toxicity. Moreover, as metal uptake and efflux are necessary steps for pathogens to invade their hosts, the role of these transporters in virulence of Enterobacteria is described.

## Metal transport systems

### Iron transport systems

**Figure 1:**
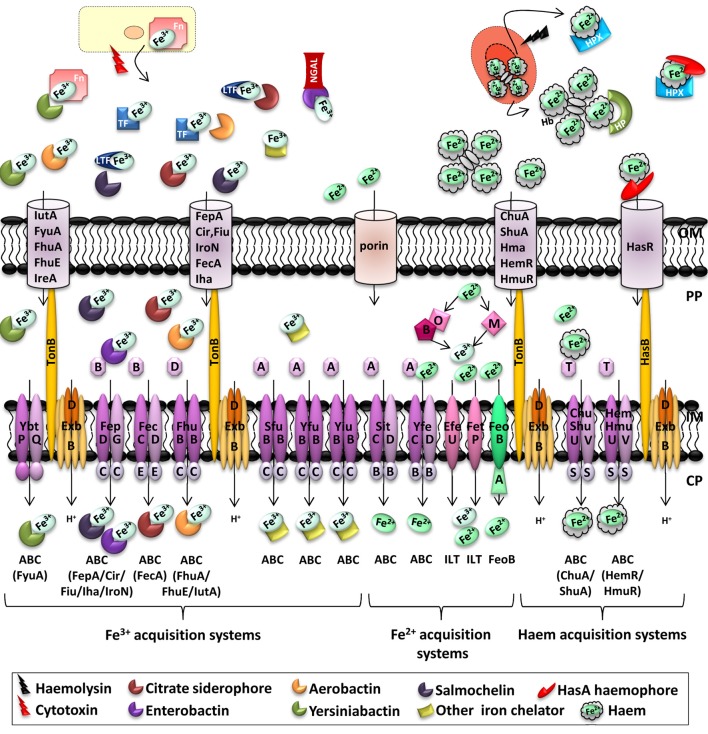
**Iron transporters in Enterobacteria and metal availability in the host during infection**. In a healthy individual, Fe^3+^ is stored intracellularly in complex with ferritin (Fn), bound by serum transferrin (TF) or bound by lactoferrin (LTF) at mucosal surfaces. In the blood, Fe^2+^ is complexed with haem, which is bound by haemoglobin (Hb) within red blood cells. During infection, haemolytic bacterial cytotoxins damage host cells, leading to the release of ferritin, while hemolytic toxins lyse erythrocytes, liberating Hb, thus, bound by haptoglobin (HP). Free haem is scavenged by haemopexin (HPX). Secreted bacterial siderophores can remove iron from transferrin, lactoferrin and ferritin, and siderophore-iron complexes are then recognized by cognate receptors at the bacterial surface. Similarly, secreted haemophores can remove haem from haemoglobin or haemopexin. Enterobacteria also possess receptors for free haem. Outer membrane receptors for haem can also transport haem from haemoglobin, and HemR/HmuR can transport haem from haemopexin and haptoglobin. Enterobactin-mediated iron acquisition can be inhibited by the innate immune protein lipocalin-2 (NGAL, Neutrophil Gelatinase-Associated Lipocalin), which binds and sequesters siderophores (Skaar, 2010; Hood and Skaar, 2012). Transporter families are indicated below transporters. One representative outer membrane receptor that transports the ligand to the periplasm represents different siderophore transporters, the identity of the outer membrane receptor being shown at the bottom under the inner membrane transporter in brackets. ABC, ATP-binding cassette; ZIP, Zrt/Irt-like protein; NRAMP, natural resistance associated with macrophage protein; OFeT, oxidase-dependent iron transporter; ILT, iron/lead transporter superfamily; FeoB, ferrous iron uptake family; OM, outer membrane; PP, periplasm; IM, inner membrane; CP, cytoplasm.

Iron is the most abundant transition metal in the host, but free ferrous iron (Fe^2+^) is extremely poorly available. Risk of infection is reduced by a strategy called “nutritional immunity,” consisting in preventing pathogens from acquiring iron. Indeed, extracellular iron, mostly present in its ferric (Fe^3+^) form, is bound to circulating transferrin. In milk, tears, saliva or in the granules of polymorphonuclear leukocytes involved in mucosal innate immune response, ferric iron is also bound to lactoferrin. These proteins display high affinity for ferric iron. Haem also constitutes an important source of iron. It contains a single Fe^2+^ atom encircled by a tetrapyrrole ring. It represents an important cofactor for haemoproteins such as haemoglobin, which is itself contained in circulating erythrocytes, rendering ferrous iron unavailable. If haemolysis occurs, free haemoglobin is bound by haptoglobin and free haem is bound to haemopexin (Johnson and Wessling-Resnick, 2012). In response to infection, a cascade of host signals leads to increased sequestration of iron. Production of Interleukin-6 by immune effector cells is triggered, leading to binding of proinflammatory cytokines to hepatocyte receptors and to increased expression of Acute Phase Proteins (APP) involved in nutritional immunity. Among these, hepcidin reduces release of iron into the circulation, ferritin promotes intracellular iron storage, and haptoglobin binds free haemoglobin (Parrow et al., 2013). Moreover, ferrous iron present in phagosomes is pumped out by Nramp1 (Hood and Skaar, 2012). Pathogenic bacteria use several strategies to acquire iron. These include import of ferrous iron by ATP- or GTP-dependent inner membrane transporters, and TonB-ExbB-ExbD dependent transport of ferric-siderophores, transferrins, haem or haem-bound proteins through specific outer membrane receptors (see Figure 1; Braun, 2001; Hood and Skaar, 2012).

#### Fe^2+^ transporters

Free Fe^2+^ is rarely present in the host, except under conditions where the redox potential or the pH are disturbed, such as ischemia caused by a trauma or following reduction of the environment by proliferating bacteria (Bullen et al., 2005). In bacteria, Fe^2+^ enters the periplasm through non-specific porins and is delivered to the cytoplasm through different transporters.

Under anaerobic-microaerophilic conditions, bacteria use the FeoB pathway (Cao et al., 2007). Members of the FeoB-family mediate transport of free Fe^2+^ across the inner membrane to the cytoplasm in a GTP-dependent manner. FeoB is located in the periplasmic membrane. It is expressed with FeoA and FeoC of recently characterized functions (Braun, 2001; Kim et al., 2013; Lau et al., 2013). FeoB is produced by the majority of *Enterobacteriaceae* Table S1; Fetherston et al., 2012; Grim et al., 2012; Hood and Skaar, 2012). In some *E. coli* strains, EfeUOB (YcdNOB) is also involved in Fe^2+^ uptake under aerobic, low-pH, low-iron conditions (Cao et al., 2007). As EfeO is able to oxidize ferrous iron, it has also been proposed that EfeU could transport Fe^3+^ (Rajasekaran et al., 2010). In *E. coli* K-12, EfeB and its paralog YfeX, widespread in Enterobacteria, may also promote iron extraction from periplasmic haem (Letoffe et al., 2009; Dailey et al., 2011). EfeUOB is also present in *Y. pestis* (Fetherston et al., 2012). An uncharacterized system called FetMP has also been identified in *E. coli* and *Y. pestis* as involved in ferrous iron uptake (Forman et al., 2010).

Inner membrane ATP-Binding Cassette (ABC) transporters can also import Fe^2+^. The ABC superfamily of transporters consists in several protein complexes which together are capable of transporting various solutes across membranes. They are composed of three or four different subunits, usually encoded by different genes grouped in an operon (Ma et al., 2009; Klein and Lewinson, 2011). The Mn^2+^/Fe^2+^ ABC-transporters of Enterobacteria include the *Yersinia* YfeABCD system and its Sit homolog identified in *S.* Typhimurium, ExPEC, *K. pneumoniae, P. mirabilis* and *Shigella* spp. Table S1; Angerer et al., 1992; Bearden and Perry, 1999; Kehres et al., 2002b; Sabri et al., 2008; Fisher et al., 2009; Himpsl et al., 2010).

#### Siderophores and low molecular weight iron chelators

Siderophores are small secreted molecules that display higher affinity for iron than the host iron-binding proteins such as transferrin and lactoferrin. They are synthesized in the cytoplasm and require specific export systems to reach the extracellular space. The export systems usually involve an inner membrane protein from the major facilitator family and an outer membrane channel protein such as TolC (Garénaux et al., 2011). In the extracellular space, iron-bound siderophores are recognized by specific receptors (Braun, 2001; Chakraborty et al., 2007). The TonB-ExbB-ExbD energy transduction system is required for transport of the ligand into the periplasmic space. While the inner-membrane-embedded ExbD-ExbB complex tranduces energy from the proton motive force of the cytoplasmic membrane, the TonB protein spans the periplasm to transfer this energy to the outer membrane receptor (Higgs et al., 2002). Then, a specific ABC-transporter mediates entry of the iron-bound siderophores through the inner membrane. A chaperone may be involved in the transfer of the iron-bound siderophore from the outer membrane receptor to an inner membrane ABC-transporter. It is the case for ferric enterobactin and aerobactin siderophores, requiring the FepB and FhuD chaperones, respectively (Koster and Braun, 1990; Chenault and Earhart, 1991). Once in the periplasm, ferric iron can be directly reduced to its ferrous state by reductases, so as to be released from the siderophores and transferred to iron-dependent proteins. As such, in some cases, apo-siderophores can be recycled to the extracellular space without the need for new siderophore biosynthesis. In other cases where the redox potential of the ferri-siderophore is too high, degradation by specific esterases is required before the reductases can release iron (Miethke et al., 2011).

Nearly all *E. coli* and *K. pneumoniae* strains produce enterobactin (Bachman et al., 2011; Garénaux et al., 2011). Enterobactin (or enterochelin), is internalized through the FepA receptor and FepCDG ABC-transporter thanks to the FepB chaperone (Chenault and Earhart, 1991). Some strains also produce a set of pathogen-specific siderophores potentially comprising salmochelins, aerobactin or yersiniabactin (Bachman et al., 2011; Garénaux et al., 2011). Salmochelins, glycosylated forms of enterobactin, are internalized through the IroN receptor and the same ABC-transporter as enterobactin, FepCDG. Aerobactin uptake involves the IutA receptor as well as FhuBC ABC-transporter, while yersiniabactin uptake occurs via the Psn/FyuA receptor and the YbtPQ ABC-transporter (Koster and Braun, 1990; Perry and Fetherston, 2011). The types of siderophores produced are dependent on the pathotypes of the strains. While ExPEC strains are able to synthesize up to four different siderophores, IPEC such as O157:H7 strains only produce enterobactin. Some non-O157 IPEC strains and commensals may also produce aerobactin (Kresse et al., 2007), and IPEC enteroaggregative strains also contain yersiniabactin- and aerobactin-encoding genes (Okeke et al., 2004). Likewise, all *Shigella* isolates produce siderophores, namely enterobactin, salmochelins or aerobactin, but the types of siderophores produced vary from one species to another (Payne et al., 2006). Salmochelins were first identified in *Salmonella*, which also produces enterobactin. Some *Salmonella* strains also produce yersiniabactin or aerobactin (Carniel, 2001; Muller et al., 2009; Izumiya et al., 2011). Siderophores produced by the different pathogenic Enterobacteria are summarized in Table S1.

Other siderophores have been identified in Enterobacteria that do not produce enterobactin. The genome of *Cronobacter* species contains a non-functional enterobactin gene cluster and a plasmid-encoded aerobactin cluster renamed cronobactin (Grim et al., 2012). Virulent *Yersinia* species are known to produce yersiniabactin as well as the alternative yersiniachelin and pseudochelin siderophores (Rakin et al., 2012). *P. mirabilis* produces proteobactin and a yersiniabactin-like siderophore (Himpsl et al., 2010).

In addition to the different siderophores produced by *E. coli* strains, it is not unusual to find more than 10 genes encoding distinct siderophore receptors in their genomes. In non-pathogenic *E. coli*, enterobactin can be internalized through 3 different TonB-dependent receptors: FepA, Cir, and Fiu (Andrews et al., 2003). The Iha TonB-dependent receptor mediates enterobactin uptake in UPEC (Leveille et al., 2006). Another TonB-dependent receptor identified in UPEC, IreA, is an additional potential siderophore transporter (Russo et al., 2001, 2002). Many Enterobacteria are able to internalize exogenous siderophores, such as the fungal siderophore ferrichrome, internalized through FhuABCD (Andrews et al., 2003). Low molecular weight iron chelators such as citrate can also be used as a source of iron. The typical ferric citrate transport system involves the FecA TonB-dependent receptor, the FecB chaperone and the FecCDE ABC-transporter at the inner membrane (Braun et al., 2003). These transport genes form the *fecABCDE* operon (Mahren et al., 2005). The *S. marcescens* SfuABC, the *K. pneumoniae* KfuABC, the *E. coli* and *Cronobacter sp* EitABC and the *Y. pestis* YfuABC and YiuABC systems transport ferric iron bound to small chelators across the cytoplasmic membrane (Angerer et al., 1990; Gong et al., 2001; Ma et al., 2005; Johnson et al., 2006; Kirillina et al., 2006; Grim et al., 2012).

This redundancy in siderophore-mediated iron acquisition systems suggests that their fundamental role lies in adaptation to different iron-limited niches in which bacteria are competing with other microorganisms in the environment or with host proteins for iron acquisition (Valdebenito et al., 2006).

#### Haem uptake

Haem represents one of the most abundant iron sources inside the host. Organization of iron in haem allows iron solubilization, but also enhances its catalytic activity by 5 to 10 times, making it an even more efficient cofactor but increasing its toxicity (Anzaldi and Skaar, 2010). Pathogens have evolved direct haem uptake systems, as well as haemophore systems.

#### Direct uptake of haem or haemoproteins

As haem is not freely available, bacteria secrete exotoxins such as haemolysins, proteases or cytolysins to release haem for direct uptake. Certain proteins of SPATE family (serine proteinase autotransporters of *Enterobacteriacae*), degrade haemoglobin to allow pathogenic bacteria to gain access to haem (Krewulak and Vogel, 2008). Haem and haemoproteins bind to specific TonB-dependent cell surface receptors, where haem contained in haemoproteins is extracted and transported to the periplasm. It is further imported by ABC transporters from the periplasm to the cytoplasm. In the cytoplasm, iron is released from haem through degradation by haem oxygenases (Hood and Skaar, 2012). *S. dysenteriae* encodes a haem transport system composed of *shuA* (coding for the haem receptor), *shuT, U* and *V* (coding for a periplasmic chaperone and an ABC-transporter) as well as *shuS* (which protects against haem toxicity by allowing its intracellular processing) and *shuWXY* coding for proteins of unknown functions (Wyckoff et al., 2005). Although the *shu* locus is only found in *S. dysenteriae*, other *Shigella* use haem as the sole iron source, suggesting another haem transport system (Payne et al., 2006). The Shu system is found in some *E. coli* strains including IPEC O157:H7 or UPEC CFT073 (Chu), *Y. pestis* (Hmu), *P. mirabilis* (Hmu) and *S. marcescens* (Hem) (Table S1), suggesting that horizontal transfer might have occurred (Thompson et al., 1999; Payne et al., 2006; Hagan and Mobley, 2009; Benevides-Matos and Biville, 2010; Himpsl et al., 2010). In UPEC, an additional haem receptor, Hma, has also been identified (Hagan and Mobley, 2009).

#### Haemophores

HasA-type haemophores were first identified in *Serratia marcescens* but are also conserved in other Gram-negative bacteria such as *Yersinia pestis* and *Y. pseudotuberculosis* (Table S1; Ghigo et al., 1997; Rossi et al., 2001). Haemophores are proteins with higher affinity for haem than haem-containing proteins such as haemopexin or myoglobin. The *S. marcescens* Haem Acquisition System (=Has) obtains haem through different steps. First, HasA haemophores are secreted through a Type I Secretion System (TISS). The SecB general chaperone is required to maintain HasA in a secretion-competent state and to facilitate its secretion. Once in the extracellular space, HasA acquires haem from the host haem carrier proteins due to its higher affinity, regardless of its redox state (Fe^2+^ or Fe^3+^). Then, haem-containing HasA is recognized by the HasR specific TonB-dependent receptor. In the presence of the HasB-ExbB-ExbD energy transduction system, interaction of haem-bound HasA with HasR leads to a conformational change allowing haem transfer to HasR and apo-HasA release in the extracellular space for recycling (Cescau et al., 2007). HasB is a TonB homolog dedicated to HasR (De Amorim et al., 2013). In *S. marcescens*, the Hem haem uptake system is active at low haem concentrations. Even lower iron concentrations are required for activation of the Has haemophore system, suggesting that these two systems might be involved in haem acquisition under different conditions (Benevides-Matos and Biville, 2010).

#### Iron export

If studies on iron homeostasis in enterobacteria mainly focus on acquisition systems, the potential role of iron efflux in virulence should also be considered. Indeed, in *S.* Typhimurium, oxidative stress caused by an iron overload is encountered after macrophage invasion. Enterobactin production and iron-citrate efflux have been shown to confer oxidative stress resistance in those conditions (Frawley et al., 2013).

### Copper transport systems

Cupric copper (Cu^2+^) is one of the most stable divalent transition metals and displays high affinity for metalloproteins. If equivalent quantities of all divalent metals were present, proteins would probably all bind copper (Waldron and Robinson, 2009). However, bacteria emerged without atmospheric O_2_. Under these conditions, copper was not soluble. As a consequence, Cu-binding proteins represent less than 0.3% of their annotated proteome. As opposed to bacteria, eukaryotic genomes evolved in the presence of copper and present a higher percentage of genes coding for Cu-binding proteins (Dupont et al., 2011). In bacteria, copper is used as a catalyzer for electron transfer reactions in some metalloenzymes involved in electron transfer reactions, such as cytochrome oxidase. It is also used as a cofactor by copper-detoxifying enzymes (Dupont et al., 2011; Hodgkinson and Petris, 2012). However, intracellular copper levels must be finely controlled, due to its toxicity. Under anaerobic conditions, copper is mainly present in the highly reactive cuprous form (Cu^1+^). Copper directly disrupts protein structures by forming thiolate bonds with iron-sulfur clusters. Degradation of iron-sulfur clusters results in an increase in free iron, which indirectly increases oxidative stress. In addition, copper interacts with polypeptide backbones and interferes with binding of some cofactors to specific amino acids (Dupont et al., 2011; Hodgkinson and Petris, 2012; Park et al., 2012). In minimal culture medium, intracellular copper in *E. coli* is low (10^−6^ M), but higher than extracellular concentration (10^−8^ M) (Outten et al., 2001).

#### Copper uptake

It is still unclear how copper enters the bacterial cytoplasm. Uncharacterized energy-independent channels such as OmpC porins probably allow passage of Cu^2+^ and Cu^1+^ through the outer membrane (Rensing and Grass, 2003). In *E. coli*, permeability to copper is reduced by the ComC outer membrane protein through an unknown mechanism. ComC expression decreases under low copper conditions to allow copper uptake. Homologs of ComC are present in many Gram-negative bacteria, suggesting that it might play an important role in copper homeostasis (Rademacher and Masepohl, 2012). Only Cu^1+^ ions enter the cytoplasm by an unknown mechanism, potentially involving Zn^2+^ uptake systems or some ATPases (Ma et al., 2009; Hood and Skaar, 2012; Nies and Herzberg, 2013). However, in the case of pathogenic Enterobacteria, copper efflux is more crucial than copper uptake. To prevent copper from competing with natural ligands of metalloproteins, unbound copper is excluded from the cytoplasm. In bacteria, all copper-dependent proteins are located in the periplasm or within the cytoplasmic membrane with binding sites in the periplasm (Ma et al., 2009; Dupont et al., 2011; Hodgkinson and Petris, 2012; Nies and Herzberg, 2013). The copper tolerance response in Gram-negative bacteria involves three different mechanisms: (1) exporting cytoplasmic copper to the periplasm using P_1B_-type ATPases, (2) detoxifying copper using the CueO multicopper oxidase, or (3) eliminating unbound periplasmic copper by exporting it or sequestering it (Dupont et al., 2011; Hodgkinson and Petris, 2012).

#### Copper export

In Gram-negative bacteria, inner membrane heavy metal pumps (P_1B_-type ATPases) export cytoplasmic copper to the periplasm. These pumps are monomers and their structure includes a group of three cytoplasmic domains responsible for the ATPase activity. The phosphorylation cycle responsible for ATPase activity induces conformational changes resulting in substrate translocation through the inner membrane (Gourdon et al., 2011; Klein and Lewinson, 2011).

**Figure 2:**
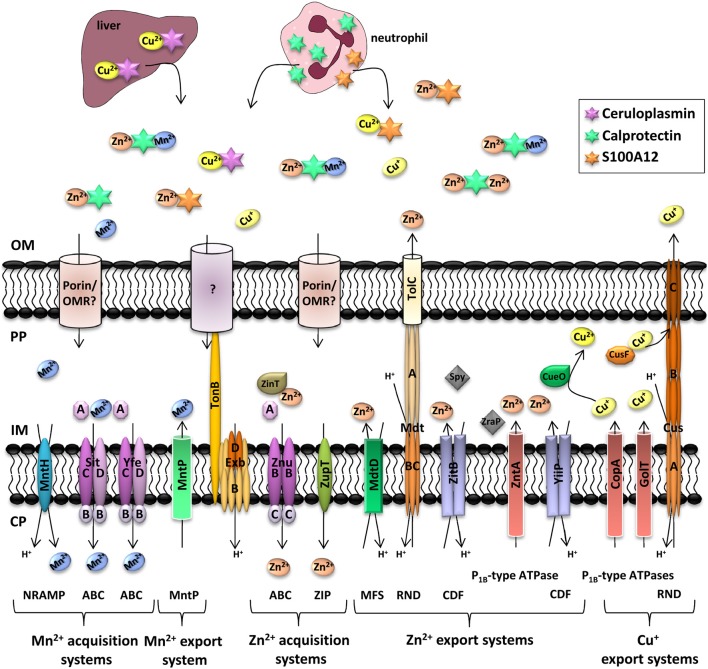
**Manganese, zinc, and copper transporters in Enterobacteria and metal availability in the host during infection**. Each microorganism possesses a different complement of transporters to ensure a good balance between import and export of metals. In deep tissues, infection leads to the recruitment of neutrophils, which deliver calprotectin and S100A12 to the infected site. To compete with host-mediated zinc and manganese sequestration, bacteria express high affinity metal transporters. It is yet unclear if uptake of Mn^2+^ and Zn^2+^ is mediated by an outer membrane receptor (OMR) coupled to the TonB/ExbB/ExbD system or by diffusion through porins. Ceruloplasmin is a multi-copper oxidase produced in the liver of healthy individuals where it binds Cu^2+^. The complex is then released in blood to bring Cu^2+^ to tissues. Following phagocytosis of bacteria in macrophages, interferon-γ induces the import of Cu^+^ inside the phagolysosome to induce bacterial killing. However, pathogenic Enterobacteria have several systems to detoxify their periplasm or cytoplasm (Hood and Skaar, 2012). Transporter families are indicated in brackets. NRAMP, natural resistance associated with macrophage protein; ABC, ATP-binding cassette; MntP, manganese transporter efflux pump family; ZIP, Zrt/Irt-like protein; MFS, Major Facilitator Superfamily; RND, resistance and nodulation; CDF, cation diffusion facilitator; OM, outer membrane; PP, periplasm; IM, inner membrane; CP, cytoplasm.

In *E. coli*, the CopA ATPase is involved in Cu^1+^ export from the cytoplasm to the periplasm (Figure 2; Arguello et al., 2011). In *Salmonella*, copper ATPases are not only required for copper resistance, but also for efficient copper availability to cupro-enzymes such as Cu/Zn SodCII under copper-limited conditions (Osman et al., 2013). They might play a role in delivering cytoplasmic copper contained in unidentified storage compounds (Nies and Herzberg, 2013). Once in the periplasm, highly reactive Cu^1+^ is detoxified through re-oxidation by the CueO multicopper oxidase to form less harmful Cu^2+^ (Dupont et al., 2011; Nies and Herzberg, 2013).

In some enterobacteria such as *E. coli*, the CusCBA heteromultimeric transport complex can transfer periplasmic copper to the extracellular milieu (Outten et al., 2001; Gudipaty et al., 2012). Using the proton-motive force, the CusCBA complex shuttles between three conformational states to act as a peristaltic pump and excrete copper (Dupont et al., 2011). The periplasmic transfer of Cu^1+^ from the P_1B_-ATPases to the CusB adaptor protein is achieved by the CusF chaperone to limit its potential toxic effects (Hodgkinson and Petris, 2012; Nies and Herzberg, 2013). This Cus system is also present in *K. pneumoniae* (Zulfiqar and Shakoori, 2012). Other chaperones have been described in some *E. coli* strains, such as plasmidic PcoC or PcoE (Espirito Santo et al., 2008).

*Salmonella* and *Yersinia* lack the CusCBA system to export Cu^1+^ from the periplasm to the extracellular milieu. Alternatively, another periplasmic protein, CueP, sequesters copper to neutralize toxicity (Pontel and Soncini, 2009; Osman et al., 2010; Dupont et al., 2011). This chaperone also helps supply copper to SodCII, (Nies and Herzberg, 2013; Osman et al., 2013). *S.* Typhimurium uses two different P_1B_-type ATPases, GolT, and CopA (Dupont et al., 2011; Nies and Herzberg, 2013). Initially described as a gold efflux system, GolT is predominantly involved in copper efflux (Osman et al., 2013). *S.* Typhimurium expresses a CueO multi-copper oxidase, also called CuiD, which buffers copper toxicity in the periplasm (Arguello et al., 2011; Hodgkinson and Petris, 2012). Periplasmic Cu^2+^ is efficiently reduced to Cu^1+^ by NADH dehydrogenase 2 and components of the respiratory chain. Re-oxidation by multicopper oxidases ensures Cu^2+^ availability for copper-dependent enzymes (Nies and Herzberg, 2013).

Iron and copper homeostasis are linked, as some siderophores interact with copper. *In vitro*, catecholate siderophores increase copper sensitivity in *E.coli*, reducing Cu^2+^ to generate toxic Cu^1+^. The CueO multicopper oxidase is essential, as it allows oxidation of catecholates to create molecules able to sequester Cu^2+^ instead of reacting with it (Grass et al., 2004). However, in *S.* Typhimurium, a *cueO* mutant does not display higher sensitivity to siderophores (Achard et al., 2010). Conversely, yersiniabactin production increases copper resistance, as it sequesters Cu^2+^ before it interacts with catecholate siderophores to produce highly toxic Cu^1+^ (Chaturvedi et al., 2012).

### Zinc transport systems

Zinc is an essential transition metal in all organisms, playing an important catalytic and structural role in a number of proteins. When relative abundance is considered, zinc represents the second most important transition metal ion in living organisms after iron. In contrast to other transition metal ions, zinc does not undergo redox reactions. Bacteria are predicted to incorporate zinc into 5–6% of all proteins (Andreini et al., 2006). Zinc plays a role in bacterial gene expression, general cellular metabolism and acts as a cofactor of virulence factors. Zinc proteins are involved in DNA replication, glycolysis, pH regulation and the biosynthesis of amino acids, extracellular peptidoglycan and low molecular weight thiols, and as a result, zinc status is linked to maintenance of the intracellular redox buffering of the cell. The apparent concentration of zinc in *E. coli* is 10^−4^ M (Outten and O'halloran, 2001). Procuring sufficient zinc to sustain growth during infection is a considerable challenge for bacterial pathogens. Serum levels of zinc are in the micromolar range, and the metal's bioavailability is further restricted because it is tightly bound to proteins. As with iron, mammals sequester zinc systematically and locally in an attempt to deprive invading pathogens of this critical micronutrient (Desrosiers et al., 2010). While zinc is an essential nutrient, excess zinc is toxic to the cell, possibly through inhibition of key enzymes and competition with other relevant metal ions (Wang and Fierke, 2013). Bacterial cells thus need to achieve a delicate balance between ensuring sufficient concentrations of zinc to fulfill essential functions while limiting concentration to prevent toxic effects. In Enterobacteria, zinc homeostasis is mediated primarily by a network of zinc influx and efflux pumps (Figure 2; Wang et al., 2012).

#### Zinc uptake

The transport of Zn^2+^ across the outer membrane is not defined in Enterobacteria. Zinc uptake across the cytoplasmic membrane is mediated by two major types of transporters: ZnuACB, which belongs to the cluster C9 family of (TroA-like) ABC transporters, and ZupT, which is a member of the ZIP (ZRT/IRT-like protein) family of transporters that are also present in eukaryotes (Hantke, 2005). ZnuACB is a high-affinity transporter whereas ZupT is a low-affinity uptake system (Hantke, 2005). Under conditions of moderate zinc availability, zinc uptake is carried out by ZupT, whereas it is carried out by ZnuACB in environments characterized by very low zinc availability.

The gene *znuA* encodes for the periplasmic, zinc-binding component of the transporter, *znuB* encodes for the transmembrane component and *znuC* encodes for the ATPase subunit. Zinc uptake mediated by the Znu system requires ATP hydrolysis by dimeric ZnuC to transport Zn^2+^ captured by ZnuA through the pore formed by a ZnuB dimer in the cytoplasmic membrane (Patzer and Hantke, 1998, 2000). In Enterobacteria, numerous studies in different strains of *E. coli*, in *S.* Typhimurium, *S.* Enteritidis, *Proteus mirabilis* or in different species of *Yersinia* have demonstrated that Δ*znuA*, Δ*znuC*, Δ*znuB* or Δ*znuACB* mutants had decreased Zn^2+^ uptake compared to the wild-type strains (Table S1; Patzer and Hantke, 1998; Campoy et al., 2002; Ammendola et al., 2007; Gunasekera et al., 2009; Sabri et al., 2009; Desrosiers et al., 2010; Nielubowicz et al., 2010; Gabbianelli et al., 2011).

In some bacterial species such as IPEC strain O157:H7 and *S.* Typhimurium, zinc uptake involves another protein, ZinT (formerly known as YodA). This protein is involved in periplasmic zinc binding under zinc-limiting conditions, and studies carried out in *S.* Typhimurium have suggested that ZinT participates in zinc uptake through ZnuACB, by a mechanism involving direct interaction with ZnuA (Petrarca et al., 2010; Gabbianelli et al., 2011).

Proteins of the ZIP family were initially identified as iron or zinc transporters in eukaryotes, but some members were subsequently shown to also transport other metals, such as manganese or cadmium. ZupT is the first characterized bacterial member of this family and was shown to be responsible for zinc uptake in *E. coli* (Grass et al., 2002). Studies on the role of ZupT on Zn^2+^ uptake have been performed in non-pathogenic *E. coli* and in the UPEC strain CFT073 (Table S1; Grass et al., 2002; Sabri et al., 2009). The *zupT* gene is constitutively expressed and ZupT can transport iron and cobalt in addition to zinc and possibly manganese. When overexpressed, ZupT can also transport copper (Grass et al., 2002, 2005).

#### Zinc export

Zinc detoxification is primarily achieved by the P_1B_-type ATPase ZntA and the cation diffusion facilitators (CDF) ZitB and YiiP (Table S1; Rensing et al., 1997; Grass et al., 2001; Wei and Fu, 2006). P_1B_-type ATPases and CDF transporters catalyze metal translocation across the inner membrane, and the substrate is transported from the cytoplasm to the periplasm (Klein and Lewinson, 2011). CDF is a ubiquitous family of metal transporters found in prokaryotes and eukaryotes. Functional analysis of YiiP and ZipT indicated that these two proteins are proton-linked antiporters that utilize the free energy derived from H^+^ influx to pump cytosolic Zn^2+^ out of the cells (Chao and Fu, 2004; Wei and Fu, 2006).

In addition to Zn^2+^, ZntA is able to transport Cd^2+^ and Pb^2+^ (Binet and Poole, 2000). It has been proposed in *S.* Typhimurium that the periplasmic C-terminal domain of ZraP, a periplasmic protein with two zinc-binding domains, facilitates modulation of transporters such as ZntA (Appia-Ayme et al., 2012). It has been suggested that ZntA is critical for the survival of *E. coli* in the presence of high zinc concentrations, while ZitB maintains zinc homeostasis under normal growth conditions, i.e., low environmental zinc stress (Rensing et al., 1997; Wang et al., 2012). Overexpression of *zitB* in *E. coli* resulted in a significant increase in zinc tolerance and reduced uptake of zinc, while overexpression of *yiiP* did not confer additional zinc resistance, and deletion of *yiiP* did not alter zinc resistance (Grass et al., 2001).

A recent study identified new zinc exporters in *E. coli*, MdtABC, a RND-type efflux pump, and MdtD, a MFS (Major Facilitator Superfamilly) transporter, as well as a periplasmic protein, Spy, involved in zinc detoxification (Table S1). It has been proposed that the role of Spy in relieving zinc stress may be to facilitate the folding and to protect the integrity of transmembrane and periplasmic transporters that function in zinc export (Wang and Fierke, 2013).

### Manganese transport systems

Manganese plays an essential role in many cellular processes including lipid, protein, and carbohydrate metabolism. It also contributes to protection against oxidative stress, is a cofactor for a number of enzymes in bacteria and other organisms, and can also contribute directly to the catalytic detoxification of reactive oxygen species (ROS) (Horsburgh et al., 2002; Kehres and Maguire, 2003). The total Mn^2+^ concentration in *E. coli* is comparable to that of Cu^+^ and is about 10-fold lower than that of Zn^2+^ (Ma et al., 2009). Like iron, manganese is found in two states, Mn^2+^ and Mn^3+^, Mn^2+^ being used by biological systems. Contrary to Fe^2+^, free Mn^2+^ is not toxic in a biological environment (Kehres and Maguire, 2003). In some bacteria, Mn^2+^ can replace the more reactive Fe^2+^ in Fe^2+^-containing proteins, reducing oxidative damage to these proteins (Hood and Skaar, 2012). The regulation of manganese homeostasis is complex and appears to overlap with peroxide defenses and iron homeostasis in bacteria (Horsburgh et al., 2002).

#### Manganese uptake

As for Zn^2+^, the mechanisms of Mn^2+^ transport across the outer membrane are not yet defined in Enterobacteria. For import across the cytoplasmic membrane, two major manganese transporters have been identified: a proton-dependent Nramp-related transport system typified by MntH and an ABC transporter typified by SitABCD and YfeABCD (Figure 2; Goswami et al., 2001; Forbes and Gros, 2003). The Nramp (Natural resistance-associated macrophage protein) transporter family was first described in plants, animals, and yeasts (Cellier et al., 1996). Mammalian Nramp1 and Nramp2 are H^+^-dependent transition metal divalent cation transporters with physiologically relevant affinities for at least Mn^2+^, Fe^2+^, and Zn^2+^ (Papp-Wallace and Maguire, 2006).

MntH (for Proton-dependent manganese transporter) has been characterized in many enterobacterial species (Table S1; Makui et al., 2000; Boyer et al., 2002; Zaharik et al., 2004; Runyen-Janecky et al., 2006; Champion et al., 2011; Perry et al., 2012). Affinity studies for Mn^2+^ have shown that the apparent K_0.5_ for Mn^2+^ uptake was 0.1 μM in *S.* Typhimurium and 0.5 to 1 μM in *E. coli*. The K_0.5_ for Fe^2+^ was about 100 μM, far higher than physiological concentrations of Fe^2+^ (Kehres et al., 2000).

High-affinity Mn^2+^ acquisition can also be mediated by ABC transporters. The SitABCD transporter, present in some pathogenic *E. coli* strains, was first described in *S.* Typhimurium (Table S1; Zhou et al., 1999). In *S.* Typhimurium, SitABCD is primarily a Mn^2+^ transporter rather than a Fe^2+^ transporter. It mediates influx of Mn^2+^ with a K_0.5_ of 0.1 μM, while it mediates influx of Zn^2+^, Cd^2+^, and Fe^2+^ with affinities of 3, 3 and 30 μM, respectively. Moreover, it operates optimally in slightly alkaline medium, whereas MntH seems to be more effective in acid medium, which suggests distinct physiological roles (Kehres et al., 2002b). In the APEC strain χ7122, the SitABCD transporter has affinities for Mn^2+^ and Fe^2+^ of about 4 and 1 μM, respectively. However, the affinities changed in accordance with the genetic background of the strain. In a *mntH* mutant, manganese was better transported than iron; in an *aroB feoB* mutant iron was better transported than manganese. SitABCD is thus a highly versatile and adaptable transporter (Sabri et al., 2006). The SitABCD transporter in *S. flexneri* or YfeABCD system in *Y. pestis* is also able to transport Mn^2+^ and Fe^2+^ (Bearden and Perry, 1999; Runyen-Janecky et al., 2006).

#### Manganese export

As manganese is essential for enzymatic catalysis and protection against oxidative stress, molecular mechanisms of manganese toxicity are not yet clear. A manganese efflux pump, MntP, has been recently described in *E. coli* K-12 (Waters et al., 2011), and this transporter seems to be present in other pathogenic Enterobacteria (Veyrier et al., 2011).

## Regulation of genes encoding metal transport systems

Genes encoding metal transporters must be tightly regulated in bacteria. Indeed, metals are toxic when present at high concentrations. Moreover, as uptake systems are not needed in all niches, it would be energetically costly to produce them anytime. They have thus to be tightly regulated to respond to the appropriate nutrient present. Regulation of metal transport systems thus, occurs primarily by metal-responsive transcriptional regulators that repress metal uptake and activate metal efflux when metal is abundant, and activate acquisition when metal is scarce. Repression of metal transport systems is thus a good way to limit metal toxicity in the presence of excess metal and to limit the energy expenses. As a pathogen encounters various environments with various metal availabilities during the course of infection, it should be able to respond precisely to generate the appropriate physiological response. Moreover, intracellular concentrations of iron, copper, zinc, and manganese are higher than extracellular concentrations of these metals. For instance, intracellular concentrations of iron, copper, and zinc in *E. coli* are 100-fold, 1000-fold and 10,000-fold higher than a chemically defined culture medium, respectively (Ma et al., 2009). Within the host, the free serum iron concentration is about 10^−24^ M, while bacteria need to maintain an intracellular iron concentration between 10^−5^ and 10^−7^ M (Garénaux et al., 2011). Bacteria thus, need highly sensitive regulatory factors responding to metal concentration to allow sufficient expression of metal uptake systems, and to ensure their physiological needs in metal nutrients.

### Regulation of iron upatke systems

The principal regulator of iron transport sytems in Enterobacteria is Fur. The Fur family of regulatory proteins is named for the *E. coli* Fe-regulated uptake repressor Fur which regulates transcription of about 90 coding and non-coding RNAs mainly related to iron homeostasis (Ma et al., 2009). As Fur also represses oxidative stress, acid resistance and virulence genes that are required for survival under infection conditions, it has been suggested that iron deprivation inside the host might constitute a signal triggering virulence factors in pathogens (Payne et al., 2006). Fur-like repressors form homodimers and display negligible affinity for the DNA operator in the apo-form, but act as transcriptional repressors by tightly binding to the DNA operator in the presence of their cognate metal ion effectors (Pennella and Giedroc, 2005; Ma et al., 2009). Fur is activated *in vitro* when iron exceeds 10^−6^ M (Waldron and Robinson, 2009). All of the above-mentioned iron acquisition systems are repressed by Fur (Table 1).

**Table 1:**
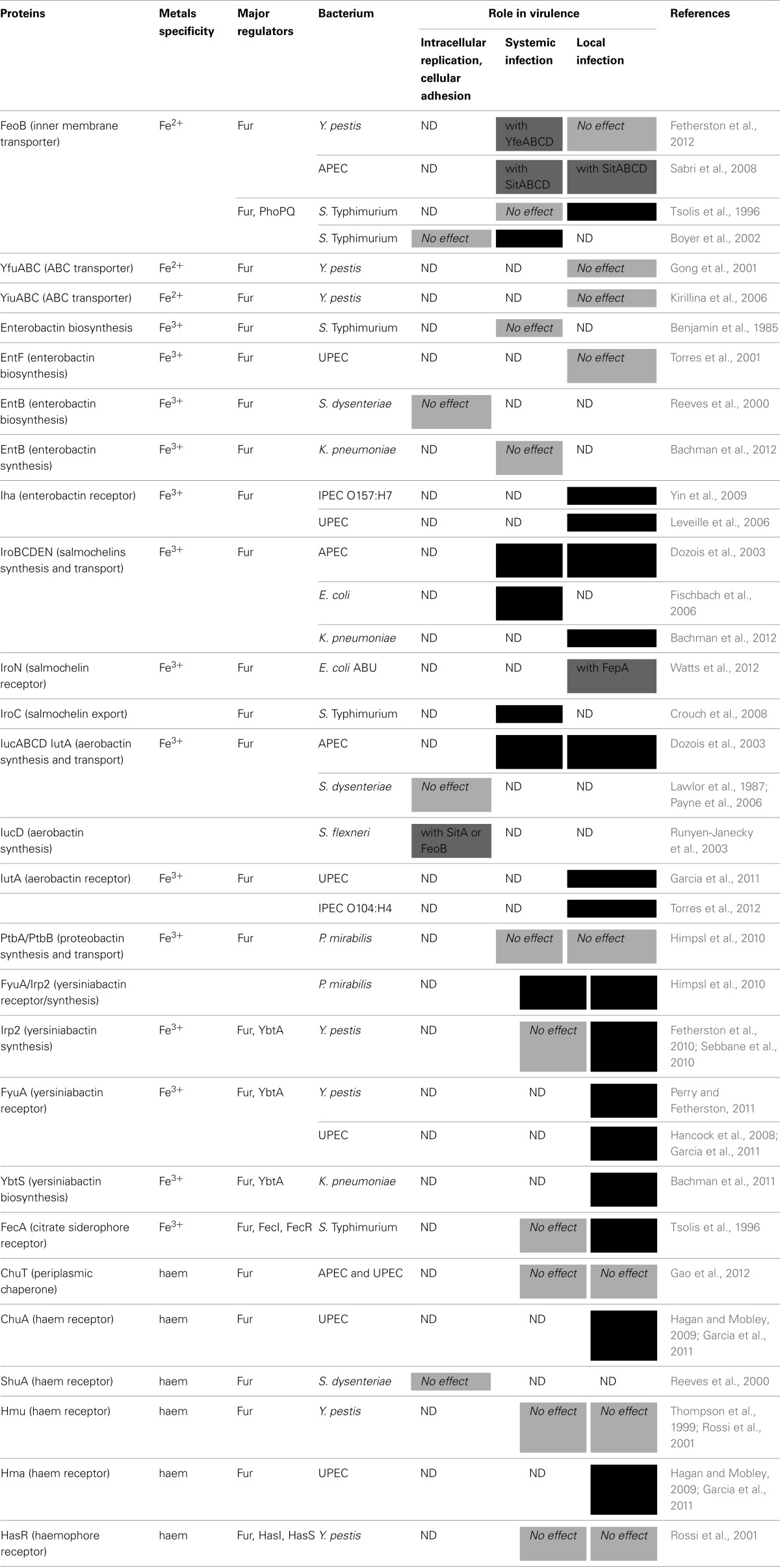
**Characterized iron transport systems involved in virulence of Enterobacteria**.

ND, Not Determined.

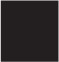
 *involved in virulence*, 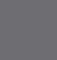 *involved with another transport system (indicated in the box)*, 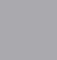 *not involved.*

Some Fur-regulated genes are overexpressed under iron-rich conditions (Braun, 2001). This might occur indirectly through the action of the Fur-regulated small RNA (sRNA) RyhB. sRNAs regulate target mRNAs by direct base pairing to positively or negatively affect their translation and stability (Storz et al., 2011). RyhB, an iron-regulated sRNA, regulates genes involved in iron metabolism. RyhB is repressed by Fur under iron-rich conditions and induced by iron starvation when Fur becomes inactive. RyhB promotes siderophore production and represses iron-using proteins (Masse and Gottesman, 2002; Masse et al., 2005). Excellent reviews focusing on the role of *ryhB* have been published recently (Salvail and Masse, 2012; Oglesby-Sherrouse and Murphy, 2013).

Regulation of synthesis and transport of some siderophores is also under the control of pathway-specific regulators. The yersiniabactin cluster is regulated by YbtA, an AraC-type transcriptional regulator. In the presence of ferri-yersiniabactin, YbtA represses its own transcription but activates transcription of yersiniabactin genes (Perry and Fetherston, 2011). Genes involved in ferric citrate and haemophore uptake are activated by a signaling cascade involving the membrane receptor as well as sigma-antisigma interactions. This signal transduction cascade is dependent on TonB (Braun et al., 2003; Biville et al., 2004; Mahren et al., 2005). In the case of the *fecABCDE* transport genes, the binding of ferric citrate to the FecA receptor creates a signal transmitted to the cytoplasmic membrane regulator FecR, which in turn activates the sigma factor FecI, directing the RNA polymerase to the promoter of the *fecABCDE* operon (Braun et al., 2003; Mahren et al., 2005). Similarly, transcription of the haemophore *has* cluster is directed by HasI sigma factor, itself inactivated by HasS antisigma factor and activated by the HasR receptor in the presence of haem-loaded haemophores (Biville et al., 2004).

Iron homeostasis does not only respond to strict iron concentrations, but also integrates a variety of signals to protect the cells against metal toxicity. Two-component regulatory systems (TCRS) regulate gene expression or protein function by responding to various environmental signals. In *Salmonella*, PhoPQ promotes Fe^2+^ uptake via the response regulator RstA that activates transcription of *feoB* in acidic pH (Choi et al., 2009). Some other TCRS do not directly regulate genes involved in metal transport, but their responses to metals can change metal availability to the cell, thus, interfering with metal transporter activation or repression. For instance, the BasS-BasR system, sensing both iron and zinc, modifies the membrane structure by inducing lipopolysaccharide remodeling, activates genes involved in membrane-associated functions (such as porins) and acts on stress-responsive regulatory proteins, such as the CsgD biofilm formation regulator, indirectly conferring resistance to metal ions (Ogasawara et al., 2012). The iron uptake transporter *efeUOB* is repressed at high pH by dephosphorylated CpxR in response to both copper and acidity (Cao et al., 2007). Interestingly, the PmrAB TCRS is the first example of a signal transduction cascade responding to extracytoplasmic Fe^3+^, and Zn^2+^ activates this system in *E. coli* but not in *S. enterica* (Chen and Groisman, 2013). Finally, the presence of oxygen also regulates iron uptake. In *S. flexneri*, anaerobic conditions activated *feoABC* while repressing genes encoding for SitABCD and the aerobactin synthesis and transport system. FeoABC was activated by the anaerobic regulators FnR and ArcA, whereas aerobactin genes were repressed by ArcA. Transcription of *fur* itself was repressed by ArcA under anaerobic conditions (Boulette and Payne, 2007).

### Regulation of copper transport systems

#### Regulation of copper uptake systems

The ComC outer membrane protein is under the control of the TetR-like ComR repressor. In the presence of high copper concentrations, Cu^2+^ binds to ComR and releases it from the *comC* promoter region, leading to its activation (Mermod et al., 2012).

#### Regulation of copper export systems

The copper ATPase CopA, the multicopper oxidase CueO and the periplasmic copper-binding protein CueP are regulated by CueR, belonging to the MerR family of regulators (Outten et al., 2000; Stoyanov et al., 2001; Osman et al., 2013). The MerR family contains nearly exclusively transcriptional activators of the expression of genes required for metal efflux or detoxification, or defense against oxidative stress and drug resistance. Both the apo- and effector-bound forms are capable of binding to their operator DNA sequences with similar affinities. However, binding of the metal ion provokes an allosteric change at the DNA-binding domain of the protein, so that only the effector-bound form can significantly optimized RNA polymerase binding and transcription initiation (O'halloran et al., 1989; Brown et al., 2003). CueR has a copper affinity of 10^−21^ M (Changela et al., 2003), and is able to induce the expression of its target genes in response to Cu^+^, Ag^+^, and Au^+^ (Ma et al., 2009; Perez Audero et al., 2010). GolS is a CueR-like sensor and the regulator of the P-type ATPase GolT (Osman et al., 2013). GolS and CueR have similar affinities for Cu^+^, but *in vivo* GolS distinguishes Au^+^ from Cu^+^ or Ag^+^ to activate its target genes (Ibanez et al., 2013; Osman et al., 2013).

The copper efflux genes *cusCFBA* are induced by the CusSR TCRS which responds to extracellular copper concentrations (Gudipaty et al., 2012). The importance of the Cus system is also dependent on the presence of oxygen. In *E. coli*, the Cus system is not required for copper resistance under aerobic conditions, in which the primary line of defense, the CueR regulon, is sufficient (Outten et al., 2001). However, in *K. pneumonia*, the Cus system is activated under both aerobic and anaerobic conditions and the activation level is higher under aerobic conditions (Zulfiqar and Shakoori, 2012). These differences might be explained by the lower capacity of *E. coli* to store copper under anaerobic conditions (Zulfiqar and Shakoori, 2012).

### Regulation of zinc transport systems

#### Regulation of zinc uptake systems

The zinc uptake system ZnuACB is regulated by Zur, belonging to the Fur family, and by SoxR, belonging to the MerR family of regulators. This system is repressed by Zn-Zur in zinc-rich environment and, under zinc depletion conditions, Zur becomes inactive, leading to the activation of *znuACB* (Li et al., 2009). Zur is able to sense zinc at concentrations as low as 10^−15^ M *in vitro* (Waldron and Robinson, 2009). SoxR responds to oxidative stress and is part of the SoxRS regulon. SoxS, activated by SoxR, activates the expression of this regulon containing numerous genes such as *znuACB* (Brown et al., 2003; Warner and Levy, 2012). A network biology approach has predicted that RybA sRNA might regulate ZupT uptake system in *E. coli* (Modi et al., 2011).

#### Regulation of zinc export systems

The ZntA export system is regulated by the MerR-like ZntR regulator. The apo-ZntR dimer binds to the promoter of *zntA* and weakly represses transcription; Zn-ZntR is a transcriptional activator (Wang et al., 2012). ZntR can also bind Pb^2+^ and Cd^2+^ to activate *zntA* expression (Binet and Poole, 2000). As Zur, ZntR has zinc affinity of 10^−15^ M *in vitro* but, *in vivo*, ZntR up-regulates *zntA* transcription in response to nanomolar intracellular concentrations of free zinc (Outten and O'halloran, 2001; Wang et al., 2012). Finally, ZntA is up-regulated under high zinc stress by ZntR to effectively lower the intracellular zinc concentration, while the other export system, ZitB is constitutively expressed to function as a first-line defense against zinc influx (Wang et al., 2012).

In Enterobacteria, responses to zinc are mediated by BasSR, BaeSR, PmrAB, and ZraSR TCRS (Yamamoto and Ishihama, 2005; Appia-Ayme et al., 2012; Chen and Groisman, 2013; Wang and Fierke, 2013). MdtABC and MdtD zinc exporters are up-regulated by BaeSR upon exposure to high concentrations of zinc (Wang and Fierke, 2013). Copper and zinc exposure lead to overexpression of Spy through regulation by CpxAR and BaeSR, respectively (Wang and Fierke, 2013), and ZraP is upregulated by ZraSR (Appia-Ayme et al., 2012).

### Regulation of manganese transport systems

#### Regulation of manganese uptake systems

In Enterobacteria, except for *Yersinia*, the DtxR-like MntR regulator is the primary sensor of manganese abundance. When bound to manganese, it represses the transcription of the uptake systems MntH and SitABCD (Patzer and Hantke, 2001; Ikeda et al., 2005; Papp-Wallace and Maguire, 2006; Runyen-Janecky et al., 2006). Of the other metal cations, only Cd^2+^ can compete efficiently for binding to MntR (Lieser et al., 2003). MntR has a 10^−5^ M affinity for manganese, value that matches the estimated 10^−5^ M intracellular concentration of this metal (Waldron and Robinson, 2009). Mn^2+^ is the most potent and most effective cation in *E. coli* and *S.* Typhimurium. In *S.* Typhimurium, Fe^2+^ repressed transcription through interaction with MntR in the absence of Fur (but this requires high extracellular concentrations of Fe^2+^) (Patzer and Hantke, 2001; Kehres et al., 2002a; Ikeda et al., 2005). In *Y. pestis*, in which MntR is absent, Fur represses YfeABCD and MntH in response to iron and manganese (Perry et al., 2012). Control of Fur repression by manganese is not specific to *Y. pestis*, as *E. coli* aerobactin and *fhuF* genes also respond to manganese in a Fur-dependent manner. However, in both cases, Fur repression through manganese binding is just specific to some promoters (Privalle and Fridovich, 1993).

MntH is activated by the LysR-type transcriptional regulator OxyR in the presence of H_2_O_2_in *S.* Typhimurium and *Shigella* (Kehres et al., 2002a; Runyen-Janecky et al., 2006). In the presence of oxidative stressors, OxyR activates genes by directly interacting with RNA polymerase to enhance initiation of transcription (Ma et al., 2009). MntH is also part of the peroxide stress response in *E. coli* (Anjem et al., 2009). It has been predicted that RybA and RyhB sRNAs might coordinately regulate *mntH* (Modi et al., 2011).

#### Regulation of manganese export systems

In addition to repressing gene expression, Mn-MntR activates the manganese efflux pump MntP (Waters et al., 2011).

### Conclusion

During metal-depletion conditions, some regulators such as Fur, Zur, or MntR (each one responding to its cognate metal) become inactive, leading to the activation of cognate metal uptake systems and entry of metal into the cell. When intracellular metal concentration increases, these regulators are activated and repress metal uptake, and other metal-bound regulators, such as ZntR, and TCRS activate efflux systems expression. This regulation thus allows to obtain sufficient nutrient for biological functions when metal is scarce, and to limit toxicity in the cell in the presence of high concentrations of metal. However, this system is organized in a highly sophisticated network, as one transporter can be regulated by its own regulator and other sensors responding to metal availability and environmental signals such as oxidative stress, pH or oxygen. This diversity of regulation for one transport system allows bacteria to sense, adapt and respond specifically and rapidly to a specific microenvironment. Moreover, one regulator can be implicated in the regulation network of several metals. For instance, Fur is able to regulate gene expression in response to iron or manganese, but is also activated by zinc (Mills and Marletta, 2005). Fur can thus be used to sense more than one metal in bacteria and the different metallo-forms of Fur control different genes by binding preferentially to different DNA sequences. Moreover, elevated levels of other metals could interfere with normal iron regulation by activating Fur inappropriately, thus, shutting down iron import and leading to iron starvation. Interestingly, the zinc and copper-dependent regulators Zur, ZntR, and CueR have very high affinities for their cognate metals, i.e., 10^−15^ and 10^−21^ M. As zinc and copper intracellular concentrations are higher from these values (10^−4^ and 10^−6^ M, respectively), this suggests that all cytoplasmic zinc and copper are bound and buffered at these low concentrations. Contrary to these regulators, Fur and MntR have higher affinities for their cognate metals (10^−6^ M for iron and 10^−5^ M for manganese, respectively), corresponding to the intracellular concentrations of these metals. This suggests that more free iron and manganese are available in the cell for other weaker metal-binding proteins, as free zinc and copper concentrations are restricting by this regulation mechanism.

## Role of metal transporters in enterobacterial virulence

Metal homeostasis plays a key role in host-pathogen interactions, as individuals suffering from iron or copper homeostasis anomalies such as thalassaemia or Menkes disease are more susceptible to infections (Vento et al., 2006; Samanovic et al., 2012). To fight infection, a first line of defense of the innate immune system is the sequestering of transition metals to specialized transfer or storage proteins, such as circulating transferrin or intracellular ferritin, which can sequester approximately 4500 Fe^3+^ ions per protein (Klein and Lewinson, 2011; Hood and Skaar, 2012). The major sources of iron available for intracellular pathogens such as *Shigella* are haem proteins and ferritin (Reeves et al., 2000). Elaborate mechanisms of transition metal limitation occur within macrophages. Following phagocytosis, bacteria are confined in the phagosome, where acidic pH, ROS and iron/manganese/zinc depletion combine to create bacteriostatic/bacteriolytic conditions. To deplete iron and manganese, the mammalian transporter Nramp1 pumps these metals out of the phagosome, while zinc is exported out of the macrophage using ZIP8 and ZnTs transporters. To acquire phagosomal metals, bacteria therefore employ high affinity transporters. Moreover, following phagocytosis of bacteria by macrophages, interferon-γ induces the import of toxic Cu^+^ inside the phagolysosome to promote bacterial killing (Kehl-Fie and Skaar, 2010; Klein and Lewinson, 2011; Hood and Skaar, 2012). Finally, S100A7, secreted by keratinocytes, inhibits microbial growth through the chelation of Zn^2+^. S100A12, expressed by neutrophils, binds both Zn^2+^ and Cu^2+^ *in vitro*, and Cu^2+^-S100A12 is involved in the generation of superoxide species. Calprotectin, also expressed by neutrophils, is able to chelate Zn^2+^ and Mn^2+^. Excellent reviews already detail the mechanisms of transition metal chelation at the host-pathogen interface (Kehl-Fie and Skaar, 2010; Hood and Skaar, 2012). The diversity of the above-mentioned metal transporters might be explained by their relative specialization to particular infection sites, i.e., intracellular niches, systemic transition or local sites of infection such as intestinal, urinary or pulmonary tracts. As such, depending on the pathogen, their importance in host defense might vary.

### Adhesion to eukaryotic cells and intracellular replication

#### Role of iron transporters

In *Shigella*, single inactivation of either the Shu haem transport system, the Sit and Feo ferrous iron transporters or enterobactin biosynthesis does not affect invasion or intracellular growth (Reeves et al., 2000; Runyen-Janecky et al., 2003). The IutA aerobactin receptor is not expressed in *Shigella* grown in HeLa cells. However, combined deletion of siderophore (IucD) and ferrous iron acquisition systems (Feo and Sit) leads to decreased growth and spreading in epithelial cells, suggesting a synergistic role of these different systems (Runyen-Janecky et al., 2003). Similarly, in *S.* Typhimurium, a single FeoB mutation does not affect intramacrophage replication, which is impaired in simultaneous absence of SitABCD, MntH, and FeoB, suggesting that ferrous iron and manganese acquisition might play a critical role in virulence (Boyer et al., 2002).

#### Role of copper transporters

The CopA ATPase, involved in export of cytoplasmic Cu^1+^ to the cytoplasm, is necessary for intramacrophage survival of *E. coli* (White et al., 2009). In *S.* Typhimurium, it is overexpressed upon phagocytosis by macrophages cells, which supports the theory that copper is accumulated in phagosomes. Deletion of *copA* or *golT* in *S.* Typhimurium has no effect on survival in cultured macrophages, whereas deletion of both results in significantly reduced survival (Achard et al., 2012; Hodgkinson and Petris, 2012). On the contrary, a *cueO* mutant shows no defect in survival in macrophages (Achard et al., 2010).

#### Role of zinc transporters

Concerning zinc, *znuA* was strongly induced in an *E. coli* O157:H7 strain adhering to Caco-2 cultured epithelial cells and a *znuA* mutant was significantly less able to adhere to Caco-2 cells in competition with the wild-type strain (Gabbianelli et al., 2011). In *S.* Typhimurium and *S.* Enteritidis, *znuA* mutants were impaired for growth in Caco-2 epithelial cells and bacteria starved for zinc displayed reduced multiplication in phagocytes (Ammendola et al., 2007).

#### Role of manganese transporters

In *Y. pseudotuberculosis*, a *mntH* mutant was defective in survival and growth in macrophages expressing functional Nramp1, but survived and replicated in macrophages deficient in Nramp. This mutant was also susceptible to killing by H_2_O_2_ when grown under manganese-limited conditions (Champion et al., 2011). In *S. flexneri*, a *mntH sitA* mutant was more sensitive to hydrogen peroxide, but not to superoxide generators. Moreover, the mutant had impaired survival in activated macrophage lines, but was able to form the same number and size plaques on Henle cell monolayers, suggesting Sit and MntH are not required for survival in this epithelial cell line. Expression of *sitA* and *mntH* was higher when *Shigella* was in Henle cells compared to LB medium (Runyen-Janecky et al., 2006). Upregulation of *sitABCD* in *Shigella* was also observed by microarray analysis in HeLa cells and human macrophage-like U937 cells (Lucchini et al., 2005). A most recent study in *S. flexneri* demonstrated that when cultured Henle cells were infected with a mixture of wild-type and *sitA* mutant strains, the *sitA* mutant was recovered in lower numbers than the wild-type strain, indicating that Sit provides an intracellular growth advantage (Fisher et al., 2009). In *S.* Typhimurium, *sitABCD* was induced *in vivo* after invasion of the intestinal mucosa (Janakiraman and Slauch, 2000). The *mntH sit* mutant of *S.* Typhimurium Keller strain was defective for replication in Nramp1^−/−^ RAW 264.7 macrophages. Overexpression of *mntH* in a *mntH sit* mutant improved the intracellular survival of the strain in macrophages (Boyer et al., 2002). In *S.* Typhimurium strain SL1344, a *mntH* mutant showed no defect in invasion of or survival in cultured HeLa or RAW 264.7 macrophages but was more susceptible to killing by H_2_O_2_. However, expression of *mntH* was induced several fold after 3h within macrophages (Kehres et al., 2000). Using the same strain, Zaharik et al. demonstrated that *sitA* and *mntH* were upregulated when strains were internalized by Nramp1-expressing macrophages (Zaharik et al., 2004). As a rule, mutants for manganese transporters impaired in their ability to replicate in macrophages were also impaired in their ability to resist oxidative stress.

### Systemic infections

#### Role of iron transporters

The FeoB ferrous iron transporter is not required for systemic infections caused by APEC or *S. enterica* infections (Tsolis et al., 1996; Sabri et al., 2008). A decrease in infection of intravenously inoculated mice was observed for *S.* Typhimurium, but the mice used in this study were Nramp^−/−^, thus, more susceptible to iron-induced oxidative stress (Boyer et al., 2002). *Y. pestis* is significantly less virulent after deletion of the *yfe, feo*, and *mntH* genes in a bubonic plague model, suggesting that iron and manganese transport are important in a subcutaneous model of infection (Fetherston et al., 2012). Pathogen-specific siderophore production is crucial for the establishment of systemic infections. Enterobactin mutants of *S.* Typhimurium are not attenuated in a systemic model of infection (Benjamin et al., 1985). Enterobactin can efficiently sequester iron from transferrin due to its particularly high affinity for ferric iron, as demonstrated in *K. pneumoniae*, in which enterobactin promotes survival in the perivascular space. However, lipocalin-2, also called NGAL or siderocalin, is an innate immune defense protein that can sequester enterobactin (Bachman et al., 2012). Chicken and quail also produce avian homologs of lipocalin-2 (Garenaux et al., 2013). Thus, production of additional siderophores that are not captured by siderocalin, are required for systemic infection by Enterobacterial pathogens (Fischbach et al., 2006). Salmochelins are involved in systemic infection caused by *S.* Typhimurium (Crouch et al., 2008). Salmochelins and aerobactin are both important for chicken systemic infection caused by APEC (Dozois et al., 2003). If yersiniabactin plays a role in bubonic plague caused by *Y. pestis*, it is not required for septicemic plague following intraveinous injection (Fetherston et al., 2010; Sebbane et al., 2010). In addition, neither the Has haemophore nor Hmu were involved in virulence in both bubonic plague or systemic infection models (Thompson et al., 1999; Rossi et al., 2001).

#### Role of copper transporters

Inside the host, infection leads to increased copper levels in the serum. This could be due to ceruloplasmin secretion by the liver during the acute-phase response. Ceruloplasmin is a serum copper-containing protein that is associated with 85% of the copper circulating inside the host (Hodgkinson and Petris, 2012). In *S.* Typhimurium, CopA and GolT ATPases are not involved in systemic infection (Achard et al., 2012; Hodgkinson and Petris, 2012). By contrast, deletion of *cueO*, resulted in decreased virulence during systemic infection (Achard et al., 2010). This is consistent with the observation that host copper deficiency increases susceptibility to infection by *S.* Typhimurium (Hodgkinson and Petris, 2012). Surprisingly, a UPEC *cueO* mutant displays a hypervirulent phenotype. Indeed, in the absence of CueO, copper-stressed cells displayed a mucoid phenotype and an aggregative behavior. This could lead to an increase in capsule production and virulence by evading the host immune response (Tree et al., 2007).

#### Role of zinc transporters

In *Y. ruckeri*, a *znuACB* mutant was unable to compete with the wild-type strain and survived poorly in rainbow trout kidney (Dahiya and Stevenson, 2010). By contrast, ZnuACB was not important for high-level infectivity and virulence of *Y. pestis* in either subcutaneous or intranasal infection models (Desrosiers et al., 2010). In *S.* Typhimurium, virulence of a *znuC* mutant was attenuated compared to the wild type strain (Campoy et al., 2002). Studies conducted with *S.* Typhimurium and *S.* Enteritidis showed similar results with *znuA* mutants (Ammendola et al., 2007).

#### Role of manganese transporters

An *yfeABCD* mutant strain of *Y. pestis* was attenuated in Nramp1^+/+^ mice following intravenous infection (Bearden and Perry, 1999). Similarly, *yfeAB, feoB yfeAB, yfe mntH* mutant strains were attenuated in a bubonic plague model (Fetherston et al., 2012; Perry et al., 2012). *Galleria mellonella* larval survival following inoculation with a *Y. pseudotuberculosis mntH* strain was significantly greater than survival following challenge with the wild-type strain (Champion et al., 2011). During intra-peritoneal competition experiments with *S.* Typhimurium, the *sitA* mutant was consistently out-competed by the wild-type strain in the spleens and livers of mice (Janakiraman and Slauch, 2000). Moreover, intravenous inoculation of *sit, mntH* and *feoB* mutant strains in Nramp1^−/−^ mice showed that the *mntH* mutant was fully virulent and the *sitABCD* mutant was markedly attenuated. The *mntH feoB, sit feoB* and *mntH sit feoB* mutants were completely avirulent (Boyer et al., 2002). Another study showed in Nramp1^+/+^ mice that the *mntH* and *sitABCD* mutants were significantly attenuated and the *mntH sit* mutant was completely avirulent (Zaharik et al., 2004). Depending on the mouse model used, MntH and SitABCD systems in *S.* Typhimurium seem to be more or less important alone but play an important combined role during infection. In *S. flexneri*, a *sitA* mutant was attenuated in a mouse lung model of virulence (Fisher et al., 2009). An APEC *sitA* mutant demonstrated reduced colonization of the lungs, liver and spleen compared to the wild-type strain. The *mntH sit* mutant demonstrated reduced persistence in blood and reduced colonization in the lungs, liver, and spleen. The *mntH* mutant was as virulent as the wild-type strain (Sabri et al., 2008). The *mntH sit* mutant strain was more sensitive to H_2_O_2_ compared to the wild-type strain (Sabri et al., 2006).

### Local infections

#### Gut colonization

***Role of iron transporters***. The FeoB ferrous iron transporter promoted colonization of the intestine by *S. enterica* in mice (Tsolis et al., 1996). Iron acquisition by siderophores also plays a particularly important role in gut colonization. Enterobactin (and other catecholate siderophores) is involved in gut colonization of the mouse by Gram-negative bacteria (Pi et al., 2012). Enterobactin is produced in large quantities by commensals or pathogens producing no other pathogen-specific siderophores, such as *E. coli* O157:H7. The Iha siderophore receptor is involved in colonization of the intestine by this enterohaemorragic *E. coli*. However, in this case, its virulence potential is related to its adhesin properties (Yin et al., 2009). Other pathogenic strains preferentially produce pathogen-specific siderophores rather than enterobactin to promote colonization (Henderson et al., 2009). As such, aerobactin is involved in mouse intestinal colonization by *E. coli* O104:H4 (Torres et al., 2012). SitA also contributes to *S*. Typhimurium colonization of the small intestine (Janakiraman and Slauch, 2000).

***Role of zinc transporters***. In *S.* Typhimurium, ZnuACB contributes to resistance against host calprotectin-mediated Zn^2+^ chelation. This transporter promotes resistance of extracellular *S.* Typhimurium to calprotectin accumulated in the host intestine following infection. Moreover, *S.* Typhimurium exploits calprotectin-mediated Zn^2+^ chelation in order to out-compete host microbiota, which is less well adapted to the zinc-limited environment in the infected intestine (Liu et al., 2012).

#### Urinary tract infections

***Role of iron transporters***. The Iha siderophore receptor is involved in bladder and kidney colonization by UPEC (Leveille et al., 2006). FyuA, the yersiniabactin receptor, promotes biofilm formation in the bladder (Hancock et al., 2008; Brumbaugh et al., 2013). In *P. mirabilis*, yersiniabactin, unlike proteobactin, also allows better fitness in the bladder and the kidneys in a coinfection model (Himpsl et al., 2010). However, as demonstrated in UPEC, yersiniabactin might be involved in copper sequestration rather than iron acquisition (Chaturvedi et al., 2012). The IutA and IroN receptors also promote bladder colonization (Garcia et al., 2011; Watts et al., 2012). On the contrary, enterobactin production is not involved in kidney colonization (Torres et al., 2001).

Haem is an important iron source in tissues. Competition assays showed that the ChuA haem receptor contributes to iron acquisition in kidneys following urinary tract infection by *E. coli* (Hagan and Mobley, 2009; Garcia et al., 2011). The Hma haem receptor is also involved in kidney colonization by UPEC (Hagan and Mobley, 2009; Garcia et al., 2011). By contrast, a *chuT* deletion mutant in mono-infections in both APEC and UPEC models had no change in virulence (Gao et al., 2012).

***Role of zinc transporters***. In competitive infections, a UPEC *zupT* mutant was not outcompeted by the wild-type strain. In contrast, the *znuA* and *znuA zupT* mutants demonstrated significantly reduced numbers in the bladders and kidneys. In single-strain infections, *znuA* and *znuA zupT* mutants were reduced in the kidneys. Moreover, the double mutant demonstrated decreased motility and less resistance to hydrogen peroxyde (Sabri et al., 2009). A UPEC *znuB* mutant exhibited a defect in biofilm formation under static conditions and in motility (Gunasekera et al., 2009). In *Proteus mirabilis*, a *znuC* mutant displayed reduced swimming and swarming motility. This mutant was outcompeted by the wild-type strain during competitive infections in urine, bladder and kidneys of mice but colonized mice as well as the wild-type during independent infections (Nielubowicz et al., 2010).

***Role of manganese transporters***. In UPEC, *mntH* and *sitABCD* mutants colonize bladder and kidneys as well as the wild type strain. However, the *mntH sit* double mutant displayed lower colonization rates in kidneys. Moreover, the *mntH* and *mntH sit* mutants were more sensitive to H_2_O_2_ and plumbagin compared to the wild-type strain (unpublished data from our laboratory).

#### Pulmonary infections

***Role of iron transporters***. Salmochelins and aerobactin play important roles in colonization of the lungs during APEC infections (Dozois et al., 2003), whereas haem uptake through the Chu system does not play a significant role (Gao et al., 2012). Salmochelins and yersiniabactin are both required for *K. pneumoniae* infections (Bachman et al., 2011, 2012). In *Y. pestis*, yersiniabactin is important for pneumonic plague but may not be critical for iron acquisition. Indeed, receptor mutants are less attenuated than synthesis mutants and it has been suggested that yersiniabactin may damage pulmonary epithelial cells or affect immune cells (Fetherston et al., 2010; Perry and Fetherston, 2011).

***Role of copper transporters***. Copper accumulation at the sites of infection, such as infected lungs by *M. tuberculosis* has been reported (Hodgkinson and Petris, 2012). As a consequence, copper tolerance might play an important role in the establishment of some pulmonary infections. However for Enterobacteria, thus far no data to support this have been reported.

***Role of manganese transporters***. Yfe and Feo systems are not essential for pneumonic plague, even though enhanced transcription of *yfe* genes was measured *in vivo* in a pneumonic plague model. However, the environment encountered by bacteria in the lungs is limited in manganese, and manganese transporters have been shown to play significant roles in other types of lung infections. Manganese requirements of *Y. pestis* during lung infection might be lower than that of other pathogens (Fetherston et al., 2012).

#### Bubonic plague

The Yfu and Yiu ABC transporters are not involved in virulence in a bubonic plague model (Gong et al., 2001; Kirillina et al., 2006). Deletion of the yersiniabactin siderophore-mediated iron acquisition system resulted in complete loss of *Y. pestis* virulence in a bubonic plague model following subcutaneous inoculation (Fetherston et al., 2010). Using a flea-to mouse infection model, it has also been established that yersiniabactin played a critical role during the early stages of the infection (Sebbane et al., 2010).

### Conclusion

Information described above has all been summarized in Tables 1, 2. All the metals described in this review are used by the host to develop defense strategies, either by starving the pathogens, or by overloading them. As a consequence, numerous sophisticated acquisition and detoxification systems have evolved in bacteria to cope with metal depletion or overload. Pathogen-specific siderophores are important for local and systemic infections. This is consistent with the fact that they can acquire iron from transferrin present in the blood and the perivascular space, and the fact that they are involved in evasion of lipocalin-2, which contributes to both systemic and mucosal innate immune defense (Chan et al., 2009). While haemophores do not contribute to virulence, other haem uptake systems seem to play a significant role. Conversely, the importance of CopA and GolT ATPases for intramacrophage survival is consistent with a localized cellular copper-mediated toxicity induced by the host under infection conditions. Recent studies have shown that iron overload is also encountered in macrophages at early stages of infection, and that catecholate (enterobactin and salmochelin) siderophores as well as iron export systems help pathogens survive in these conditions (Achard et al., 2013; Frawley et al., 2013). By contrast, uptake systems described for zinc and manganese are important for pathogens at all infection sites. Yet unidentified systems involved in metal acquisition from host proteins such as calprotectin might be involved, and the requirement or stringency for zinc and manganese acquisition may vary for different sites of infection.

**Table 2:**
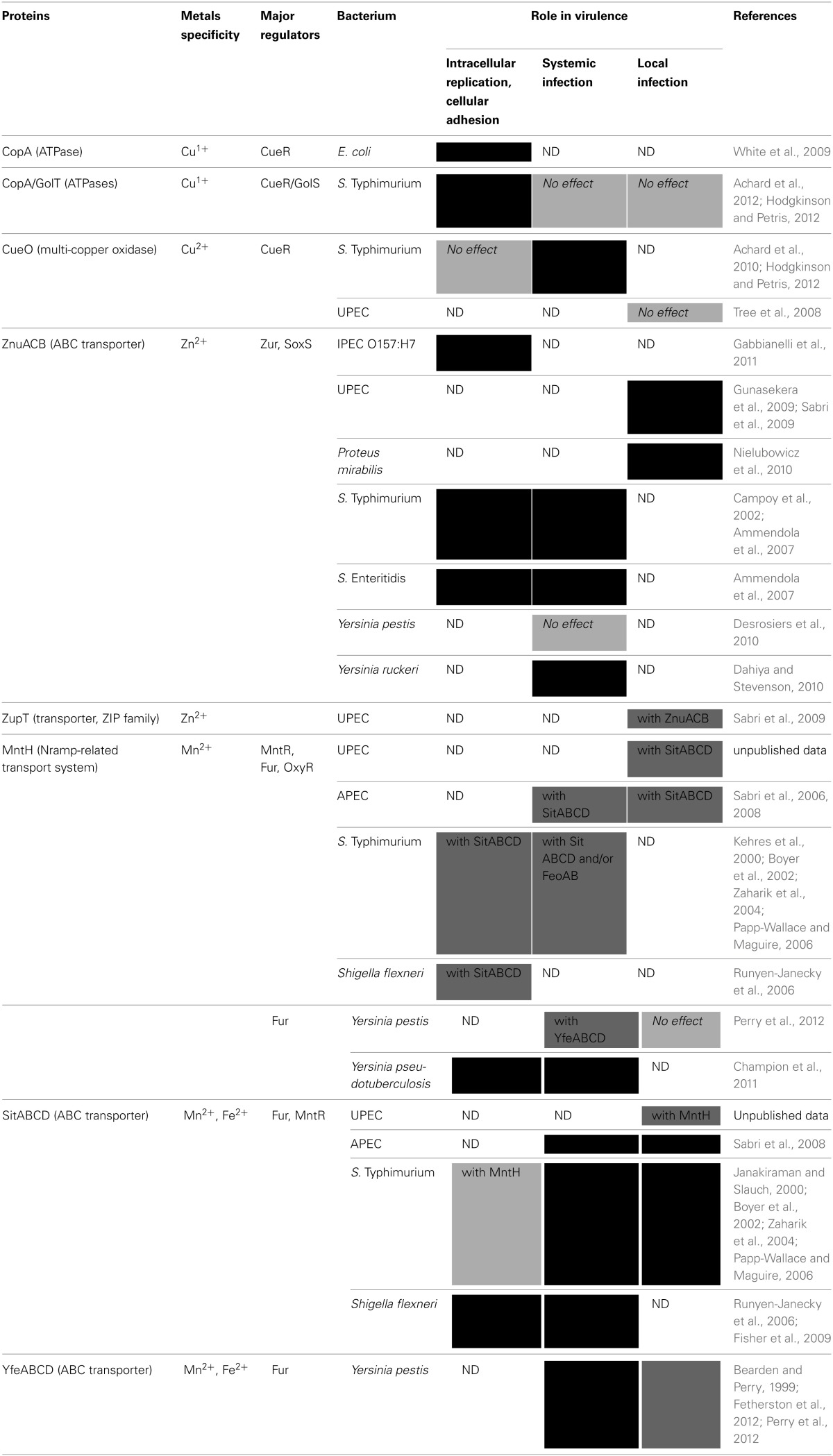
**Characterized copper, zinc and manganese transport systems involved in virulence of Enterobacteria**.

ND, Not Determined.

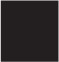
 *involved in virulence*, 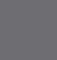 *involved with another transport system (indicated in the box)*, 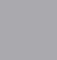 *not involved.*

## Metal acquisition within the host: a battle in which pathogenic bacteria have the upper hand?

To fight bacterial infections, the first line of defense of the host is to restrict access to essential metals, a process termed nutritional immunity. This process allows sequestration of essential metals by host proteins such as ferritin, haemoglobin or transferrin for iron, calprotectin for zinc and manganese, and ceruloplasmin for copper. In addition to extracellular metal restriction mechanisms, host cells can also deplete metals from inside phagosomes. All together, host metal sequestration should limit metal availability to invading pathogens, which should reduce their capacity to replicate and cause infections. However, pathogenic bacteria have acquired multiple mechanisms to counteract this line of defense. First of all, enterobacterial pathogens possess highly specialized and diversified regulation systems that sense intracellular metal concentrations. When a nutrient metal is lacking, these regulators (Fur, MntR, CueR, Zur…) control expression of uptake systems and repress such systems when these metals are replete. Moreover, the regulation of bacterial export systems also allows bacteria to expel metals that are in excess from the cell and hence protect the bacterial cell from metal toxicity. These regulation systems, which respond to specific concentrations of metals, allow the good pattern of expression of metal transport systems to respond to a specific microenvironment. This ensures sufficient metal uptake to allow replication while limiting metal availability in the bacterial cytoplasm to prevent toxicity. Due to the complexity of this metal regulation network, the distinctive functions of each component in the different niches encountered by pathogens during their infection cycle is still unclear. Behind this sophisticated metal regulatory network, pathogenic Enterobacteria also possess diversified metal transport systems to evade nutritional immunity. To obtain metals sequestered by host proteins, pathogenic bacteria are able firstly to produce several uptake systems for a same cognate metal. Secondly, most of these metal uptake systems have high affinity for their cognate metal, which thus, allows for example siderophores to compete with iron sequestration by host proteins such as transferrin. The diversity of bacterial metal transport systems is an important mechanism to ensure sufficient metal uptake and to adapt to different niches inside the host.

A second line of defense developed by the host against infection is to produce proteins that can specifically sequester bacterial iron-uptake systems. Lipocalin-2 is able to bind enterobactin, a siderophore secreted by many Enterobacteria. This second mechanism should represent an effective way to defend against enteric infections. However, enterobacterial pathogens have also evolved to trump lipocalin-2, by producing other types of siderophores that escape lipocalin-2 sequestration.

Finally, the multiplicity of systems produced by pathogenic Enterobacteria to regulate and transport metal nutrients illustrates why these systems are so important for their pathogenesis. Thanks to specific regulation mechanisms, they are able to sense precisely which concentrations and which types of metal are available in the specific niche they infect. They can thus adapt and respond efficiently by activating or repressing uptake or export systems corresponding to metal availability. As the type of metal and its bioavailability differs depending on the host tissue or cellular environment, this may explain why a transport system may be important for virulence of some strains while the same system may not be required for virulence in another strain associated with infection at a different tissue site. For instance, while haem uptake is important in the establishment of UTIs by UPEC (Hagan and Mobley, 2009; Garcia et al., 2011), such systems are of limited importance in bubonic plague caused by *Y. pestis* (Rossi et al., 2001). Overall, pathogenic Enterobacteria seem to have an edge over host defenses and can cause important infections due to metal acquisition. As pathogenic bacteria use tightly controlled regulation systems to respond and adapt to metal nutrient availability, it would be of interest to further elucidate how metal homeostasis is temporally regulated in bacterial cells within the host. This could allow us to specifically target and limit metal sensing by bacterial pathogens. Reducing the bacterial response to nutritional immunity, could lead to dysfunctional metal homeostasis and novel approaches to prevent and treat infections.

## Conclusion

Pathogenic bacteria encounter various metal-related stresses during infection or colonization of hosts, whether by metal starvation through metal chelation by host proteins, or by exposure to metal toxicity, for example with Cu^+^. Moreover, they have to sense metal levels to prevent metal toxicity at high concentrations. Bacteria have therefore developed very sophisticated transport systems for each metal to ensure sufficient uptake while activating efficient export of such metals if they are in excess. As these metals are essential co-factors for bacterial physiology and growth, it is not surprising that metal transporters are implicated in the virulence of pathogenic Enterobacteria. In addition, as each pathogen encounters various host environments and differences in metal availability, a specific transporter may be important for virulence of one bacterial strain, but may not be required for another strain in a distinct host species or site of infection. Historically, empirical disruption of copper homeostasis has constituted a basic hygiene measure for thousands of years (Samanovic et al., 2012). Copper surfaces, copper nanoparticles, use of copper in food supplementation or copper sprays are still used in construction and agriculture. However, this has already resulted in selection for copper-resistant strains (Dupont et al., 2011). In the last decades, a better understanding of the pathogen-specific systems involved in metal homeostasis has allowed the development of vaccination strategies or therapies. Indeed, since metal uptake systems require specific surface receptors that are exposed on the outer membrane, such receptors if immunogenic could provide targets for protective vaccines or serve as a port of entry for therapeutic molecules. Recent studies have shown that mono or multivalent receptor vaccines induce good immune responses and protect against diseases (Alteri et al., 2009; Wieser et al., 2010; Brumbaugh et al., 2013). Moreover, immunization using attenuated bacterial strains lacking metal transport systems may efficiently protect against infection. For example a *S.* Typhimurium Δ*znuACB* mutant strain has been shown to confer good mucosal protection against salmonellosis in mice and pigs (Pasquali et al., 2008; Pesciaroli et al., 2011, 2013; Gradassi et al., 2013). In addition, some bacterial strains produce antibacterial molecules that are recognized by specific siderophore receptors present on competing bacteria. For instance, microcin E492 produced by *K. pneumoniae* enters *E. coli* through its enterobactin receptors (Destoumieux-Garzon et al., 2006). Creating siderophore analogs as “Trojan horses” can be used for the development of novel antimicrobials (Miller et al., 2009). Pesticin, produced by *Y. pestis*, is known to enter through the yersiniabactin FyuA receptor. Based on this observation, a phage lysin active against Gram-negative pathogens has been engineered (Lukacik et al., 2012). A recent study also demonstrated that the probiotic *E. coli* Nissle strain reduces *S*. Typhimurium intestinal colonization by competing for iron, as this strain possesses more siderophores than *Salmonella* (Deriu et al., 2013). Future characterization of transition metal transport processes and their regulation, and determination of how cellular metal content varies and is controlled in pathogenic bacteria will further elucidate new prospects on vaccination or therapeutic development against Enterobacteria and other important bacterial pathogens.

### Conflict of interest statement

The authors declare that the research was conducted in the absence of any commercial or financial relationships that could be construed as a potential conflict of interest.
