# Supplementary material for: Iron, copper, zinc, and manganese transport and regulation in pathogenic Enterobacteria: correlations between strains, site of infection and the relative importance of the different metal transport systems for virulence
Source: Front Cell Infect Microbiol. 2013 Dec 5;3:90. doi: 10.3389/fcimb.2013.00090 (PMC3852070; doi:10.3389/fcimb.2013.00090)
Supplement: Supplementary file 1 [file Data_Sheet_1.PDF]

Supplementary Table 1. Iron, copper, zinc and manganese transport systems in Enterobacteria.

	Escherichia coli	*Salmonella enterica sp*	*Klebsiella pneumoniae*	*Shigella sp*	*Yersinia sp*	*Serratia marcescens*	*Proteus mirabilis*	*Cronobacter sp*
Ferrous iron transport								
FeoB	- F[a] (non-pathogenic) (Kammler et al., 1993)<br>- F[b] (APEC) (Sabri et al., 2008)<br>- BP (All)	- F[a,b] (*S.* Typhimurium) (Tsolis et al., 1996)<br>- BP (All)	F[c] (Hung et al., 2012)	- F[a,b] (*S. flexneri*) (Runyen-Janecky et al., 2003)<br>- BP (All)	- F[a,b] (*Y. pestis*) (Fetherston et al., 2012)<br>- BP (All)	BP	P (Himpsl et al., 2010)	P (All) (Grim et al., 2012)
EfeUOB (YcdNOB)	- F[a] (O157:H7 IPEC)<br>- NF (non-pathogenic) (Cao et al., 2007)<br>- BP (All)	BA (All)	BP	BP (All)	P (All) (Forman et al., 2010)	BP	BA	BP (All)
YfeX	- F[a] (non-pathogenic) (Letoffe et al., 2009)<br>- BP (All)	BP (All)	BP	BP (All)	BP (All)	BP	BP	BP (All)
FetMP	- F[a] (UPEC) (Koch et al., 2011)<br>- BP (ExPEC)<br>- BA (IPEC and non-pathogenic)	- BP (*S.* Typhimurium)<br>- BA (All others)	BA	BA (All)	BP (All)	BA	BA	BA (All)
SitABCD, YfeABCD	Sit<br>- F[a,b] (APEC)	Sit<br>- F[a,b] (*S.*	Sit<br>P	Sit<br>- F[b] (*S.*	Yfe<br>- F[a,b] (*Y. pestis*)	BP	Sit<br>P	BA (All)

	(Sabri et al., 2006; Sabri et al., 2008)<br>- P (enteroinvasive IPEC)<br>- A (other IPEC)<br>(Runyen-Janecky et al., 2003)<br>- BP (ExPEC)<br>- BA (non-pathogenic)	Typhimurium)<br>(Boyer et al., 2002; Kehres et al., 2002)<br>- BP (All)	(Hsieh et al., 2008)	*flexneri*)<br>(Runyen-Janecky et al., 2003)<br>- BP (All)	(Bearden and Perry, 1999)<br>- BP (All)		(Himpsl et al., 2010)	
Ferric iron acquisition by siderophore synthesis								
Enterobactin	- F[a] (non-pathogenic)<br>(Cox et al., 1970)<br>- BP (All)	- F[a] (*S.* Typhimurium)<br>(Pollack et al., 1970)<br>- BP (All)	F[a] (Perry and San Clemente, 1979)	- F[a] (*S. sonnei*)<br>(Perry and San Clemente, 1979)<br>- BP (*S. dysenteriae*; deletions in *S. flexneri* and *S. boydii*)	BA (All)	F[a] (Angerer et al., 1992)	BA	BP (All)
Salmochelins	- F[a,b] (UPEC and APEC)<br>(Dozois et al., 2003; Hantke et al., 2003)<br>- BP (ExPEC)<br>- BA (non-pathogenic and IPEC)	- F[a,b] (*S.* Typhimurium)<br>(Hantke et al., 2003; Crouch et al., 2008)<br>- BP (All)	F[a,b] (Hsieh et al., 2008; Bachman et al., 2012)	- P (*S. dysenteriae*)<br>- A (All others)<br>(Payne et al., 2006)	BA (All)	BA	BA	BA (All)

Aerobactin	- F[a,b] (ExPEC) (Warner et al., 1981; Dozois et al., 2003)<br>- BP (ExPEC, IPEC non O157:H7 and non-pathogenic)	- P (emerging *S.* Typhimurium) (Izumiya et al., 2011)<br>- BA (All others)	F[a,b] (Hsieh et al., 2008)	- P (*S. flexneri*) (Marolda et al., 1987)<br>- P (*S. sonnei* and *boydii*)<br>- A (*S. dysenteriae*) (Payne et al., 2006)	- NF (*Y. pseudotuberculosis* and *Y. pestis*) (Forman et al., 2010)<br>- BA (*Y. enterocolitica*)	BA	BA	- F[a] (*C. sakazakii*)<br>- P (All) (Grim et al., 2012)
Yersiniachelin	BA (All)	BA (All)	BA	BA (All)	- F[a] (*Y. pestis*) (Podladchikova et al., 2012)<br>- P (*Y. pestis* and *pseudotuberculosis*)<br>- A (*Y. enterocolitica*) (Rakin et al., 2012)	BA	BA	BA (All)
Yersiniabactin	- F[a,b] (ExPEC) (Schubert et al., 2000; Garcia et al., 2011)<br>- BP (ExPEC, enteroaggregative IPEC and non-pathogenic)<br>- BA (other IPEC)	- F[a] (*some S. enterica* subspecies III and VI)<br>- A (*S.* Typhimurium, *S.* Enteritidis and *S.* Typhi) (Oelschlaeger et al., 2003)	F[a,b] (Hsieh et al., 2008)	BA (All)	- F[a,b] (*Y. pestis*) (Bearden et al., 1997)<br>- BP (All)	BA	BA	BA (All)

Pseudochelin	BA (All)	BA (All)	BA	BA (All)	- P (*Y. pestis* and *Y. pseudotuberculosis*)<br>- A (*Y. enterocolitica*) (Rakin et al., 2012)	BA	BA	BA (All)
Yersiniabactin-like siderophore	BA (All)	BA (All)	BA	BA (All)	BA (All)	BA	F[a,b] (Himpsl et al., 2010)	BA (All)
Proteobactin	BA (All)	BA (All)	BA	BA (All)	BA (All)	BA	F[a] (Himpsl et al., 2010)	BA (All)
Additional TonB-dependent exogenous siderophore receptors								
CirA	- F[a] (non-pathogenic) (Hantke, 1990)<br>- BP (All)	BP (All)	BP	BP (All)	BP (All)	BP	P (Himpsl et al., 2010)	BA (All)
Fiu	- F[a] (non-pathogenic) (Hantke, 1990)<br>- BP (All)	BA (All)	BP	BP (All)	BA (All)	BA	BA	BA (All)
Iha	- F[a,b] (APEC) (Leveille et al., 2006)<br>- BP (ExPEC and IPEC)<br>- BA (non-pathogenic)	BA (All)	BA	- BP (*S. boydii*)<br>- BA (All others)	BA (All)	BA	BA	BA (All)
IreA	- F[b] (UPEC) (Russo et al., 2001)	BA (All)	BA	BA (All)	BA (All)	BA	P (Himpsl et al., 2010)	BA (All)

	- BP (ExPEC)<br>- BA (IPEC and non-pathogenic)							
FhuA	- F[a] (non-pathogenic) (Braun et al., 2003)<br>- BP (All)	BP (All)	BP	BP (All)	BA (All)	BP	P (Himpsl et al., 2010)	P (All) (Grim et al., 2012)
FepA	- F[a] (non-pathogenic) (Chakraborty et al., 2003)<br>- BP (All)	BP (All)	BP	BP (All)	BA (All)	BP	BA	P (All) (Grim et al., 2012)
YncD	BP (All)	BP (All)	BP	BP (All)	BA (All)	BP	BA	P (All) (Grim et al., 2012)
FoxA	BA (All)	BA (All)	BP	BA (All)	- F[a] (*Y. enterocolitica*) (Deiss et al., 1998)<br>- BA (All others)	BA	BA	P (All) (Grim et al., 2012)
FhuE	- F[a] (non-pathogenic) (Sauer et al., 1990)<br>- BP (All)	BP (All)	BA	BP (All)	P (All)	BA (All)	BA	P (All) (Grim et al., 2012)
Ferric iron transport by other low molecular weight chelators (citrate,…)								
FecABCDE	- F[a] (non-pathogenic) (Mahren et al., 2005)<br>- BP (All)	BA (All)	F[a] (Mahren et al., 2005)	- F[a] (*S. flexneri*) (Luck et al., 2001)<br>- BP (*S. sonnei* and *S.*	BA (All)	BA	BA	BA (All)

				dysenteriae)<br>- BA (*S. boydii*)				
Ferric iron ABC transporters	Eit<br>- P (APEC) (Johnson et al., 2006)<br>- BA (All others)	BA (All)	Kfu<br>F[a,b] (Ma et al., 2005)	BA (All)	Yiu and Yfu<br>- F[a] (*Y. pestis*) (Kirillina et al., 2006)<br>- Yiu BP (*Y. pestis* and *Y. pseudotuberculosis*)<br>- Yfu BP (All)	Sfu<br>F[a] (Angerer et al., 1992)	BA	Eit<br>P (All) (Grim et al., 2012)
Direct haem uptake								
Shu, Chu, Hmu, Hem	Chu<br>- F[a] (O157 :H7 IPEC) (Torres and Payne, 1997)<br>- F[a,b] (UPEC) (Hagan and Mobley, 2009)<br>- BP (ExPEC, enterohaemorrhagic IPEC)<br>- BA (non-pathogenic, other IPEC)	BA (All)	BA	Shu<br>- F[a] (*S. dysenteriae*) (Wyckoff et al., 2005)<br>- BA (All others)	Hmu<br>- F[a] (*Y. pestis*) (Thompson et al., 1999)<br>- BP (*Y. pseudotuberculosis*)<br>Hem<br>- F[a] (*Y. enterocolitica*) (Stojiljkovic and Hantke, 1992)	Hem<br>F[a] (Benevides-Matos and Biville, 2010)	Hmu<br>P (Himpsl et al., 2010)	BA (All)
Hma	- F[a,b] (UPEC) (Hagan and Mobley, 2009)<br>- BP (ExPEC,	BA (All)	BA	BA (All)	BA (All)	BA	BA	BA (All)

	enterohaemorrhagic IPEC)<br>- partial in non-pathogenic							
Haemophores								
Has	BA (All)	BA (All)	BA	BA (All)	- F[a] (*Y. pestis*) (Rossi et al., 2001)<br>- BP (All)	F[a] (Ghigo et al., 1997)	BA	BA (All)
Copper uptake								
ComC	- F[a] (non-pathogenic) (Mermod et al., 2012)<br>- BP (All)	BP (All)	BP	BP (All)	BA (All)	BA	BA	BP (All)
Copper export								
CopA	- F[a] (non-pathogenic) (Rensing et al., 2000)<br>- BP (All)	- F[a,b] (*S.* Typhimurium) (Espariz et al., 2007; Achard et al., 2012)<br>- BP (All)	P (Zulfiqar and Shakoori, 2012)	BP (All)	BP (All)	F[a] (Williamson et al., 2006)	BP	BP (All)
GolT	- A (non-pathogenic) (Espariz et al., 2007)<br>- BA (All)	- F[a,b] (*S.* Typhimurium) (Espariz et al., 2007; Achard et al., 2012)	BA	BA (All)	BA (All)	BA	BP (pseudogene)	BA (All)

		- A (*S.* Typhi) (Espariz et al., 2007)<br>- BP (All others)						
CueO (CuiD)	- F[a] (non-pathogenic) (Tree et al., 2005)<br>- BP (All)	- F[a,b] (*S.* Typhimurium) (Espariz et al., 2007; Achard et al., 2010)<br>- BP (All)	F[a] (Li et al., 2008)	BP (All)	BP (All)	BP	BP	BP (All)
CusCBA	- F[a] (non-pathogenic) (Outten et al., 2001)<br>- BP (All)	- A (*S.* Typhimurium, *S.* Typhi) (Espariz et al., 2007)<br>- BP (*S.* Heidelberg and *S.* Cubana)<br>- BA (All others)	F[a] (Zulfiqar and Shakoori, 2012)	- BP (*S. sonnei* and *S. flexneri*)<br>- BA (*S. boydii* and *S. dysenteriae*)	BA (All)	BP	BA	BP (All)
PcoABCDRS	- F[a] (ExPEC) (Brown et al., 1995)<br>- BP (APEC, enteroaggregative and enterotoxigenic	- BP (*S.* Heidelberg and *S.* Cubana)<br>- BA (All others)	BP	BA (All)	BA (All)	BP	BA	BP (All)

	IPEC)<br>- BA (All others)							
CueP	BA (All)	- F[a] (*S.* Typhimurium)<br>(Osman et al., 2010)<br>- BP (All)	BA	BA (All)	BP (All)	BA	BA	BA (All)
Zinc uptake								
ZnuACB	- F[a] (non-pathogenic)<br>(Patzer and Hantke, 1998)<br>- F[a,b] (UPEC, IPEC)<br>(Sabri et al., 2009; Gabbianelli et al., 2011)<br>- BP (All)	- F[a,b] (*S.* Typhimurium and *S.* Enterica)<br>(Ammendola et al., 2007)<br>- BP (All)	BP	BP (All)	- F[a] (*Y.pestis*)<br>(Desrosiers et al., 2010)<br>- BP (All)	BP	F[a,b]<br>(Nielubowicz et al., 2010)	BP (All)
ZinT	- F[a] (non-pathogenic)<br>(Kershaw et al., 2007)<br>- BP (All)	- F[a] (*S.* Typhimurium)<br>(Petrarca et al., 2010)<br>- BP (All)	BP	BP (All)	BA (All)	BP	BA	BP (All)
ZupT	- F[a] (non-pathogenic)<br>(Grass et al., 2002)<br>- F[a,b] (UPEC)<br>(Sabri et al., 2009)	BP (All)	BP	BP (All)	BA (All)	BP	BA	BP (All)

	- BP (All)							
Zinc export								
ZntA	- F[a] (non-pathogenic) (Rensing et al., 1997)<br>- BP (All)	- F[a] (*S.* Typhimurium) (Perez et al., 2006)<br>- BP (All)	BP	BP (All)	BP (All)	BP	F[a] (Rensing et al., 1998)	BP (All)
ZitB	- F[a] (non-pathogenic) (Grass et al., 2001)<br>- BP (All)	BP (All)	BP	BP (All)	BP (All)	BP	BA	BP (All)
YiiP	- F[a] (non-pathogenic) (Wei and Fu, 2006)<br>- BP (All)	BP (All)	BP	BP (All)	BP (All)	BP	BA	BP (All)
ZraP	- F[a] (non-pathogenic) (Noll et al., 1998)<br>- BP (All)	- F[a] (*S.* Typhimurium) (Appia-Ayme et al., 2012)<br>- BP (All)	BP	BP (All)	BA (All)	BA	BA	BA (All)
MdtABC	- F[a] (non-pathogenic) (Wang and Fierke, 2013)<br>- BP (All)	BP (All)	BP	- BA (*S. dysenteriae*)<br>- BP (All others)	BP (All)	BP	BP	BP (All)
MdtD	- F[a] (non-pathogenic) (Wang and Fierke,	BP (All)	BP	BP (All)	BP (All)	BP	BA	BP (All)

	2013)<br>- BP (All)							
Spy	- F[a] (non-pathogenic)<br>(Wang and Fierke, 2013)<br>- BP (All)	- F[a] (*S.* Typhimurium)<br>(Appia-Ayme et al., 2012)<br>- BP (All)	BP	BP (All)	BA (All)	BP	BA	BP (All)
Manganese uptake								
MntH	- F[a] (non-pathogenic)<br>(Makui et al., 2000)<br>- F[a,b] (APEC)<br>(Sabri et al., 2006; Sabri et al., 2008)<br>- BP (All)	- F[a,b] (*S.* Typhimurium)<br>(Kehres et al., 2000; Boyer et al., 2002)<br>- BP (All)	BP	- F[a,b] (*S. flexneri*)<br>(Runyen-Janecky et al., 2006)<br>- BP (All)	- F[a,b] (*Y. pestis*)<br>(Perry et al., 2012)<br>- BP (All)	BP	BA	BP (All)
SitABCD, YfeABCD	Sit<br>- F[a,b] (APEC)<br>(Sabri et al., 2006; Sabri et al., 2008)<br>- P (enteroinvasive IPEC)<br>- A (other IPEC)<br>(Runyen-Janecky et al., 2003)<br>- BP (ExPEC)<br>- BA (non-pathogenic)	Sit<br>- F[a,b] (*S.* Typhimurium)<br>(Boyer et al., 2002; Kehres et al., 2002)<br>- BP (All)	Sit<br>P<br>(Hsieh et al., 2008)	Sit<br>- F[b] (*S. flexneri*)<br>(Runyen-Janecky et al., 2003)<br>- BP (All)	Yfe<br>- F[a,b] (*Y. pestis*)<br>(Bearden and Perry, 1999)<br>- BP (All)	BP	Sit<br>P<br>(Himpsl et al., 2010)	BA (All)
Manganese export								
MntP	- F[a] (non-pathogenic)	BP (All)	BP	BP (All)	BP (All)	BP	BP	BP (All)

	(Waters et al., 2011)<br>- BP (All)							

P, present in the genome, as described in the cited publication; A, not found in the genome, as described in the cited publication; F, experimentally characterized as functional; NF, non-functional according to experimental data; BP, presence determined by bioinformatics (Blastn analyses); BA, absence determined by bioinformatics (Blastn analyses). Bioinformatics analyses were performed when a system was not described in the literature for a particular species.

[a], functionality refers to a demonstrated role in metal transport; [b], functionality refers to a demonstrated role in virulence; [c], functionality refers to a characterized structure of proteins.

“All” means that a system is represented in some strains belonging to each of the species tested. “All” for *E. coli* refers to ExPEC, IPEC and non-pathogenic strains. “All” for *Salmonella enterica* refers to all serovars of the subspecies *enterica*. “All” for *Shigella* refers to *S. flexneri*, *S. boydii*, *S. dysenteriae* and *S. sonnei* species. “All” for *Yersinia* refers to *Y. pestis*, *Y. enterocolitica* and *Y. pseudotuberculosis* species. “All” for *Cronobacter* refers to *C. turicensis* and *C. sakazakii* species.

References

Achard, M.E., Stafford, S.L., Bokil, N.J., Chartres, J., Bernhardt, P.V., Schembri, M.A., Sweet, M.J., and Mcewan, A.G. (2012). Copper redistribution in murine macrophages in response to *Salmonella* infection. *Biochem J*. 444**,** 51-57.

Achard, M.E., Tree, J.J., Holden, J.A., Simpfendorfer, K.R., Wijburg, O.L., Strugnell, R.A., Schembri, M.A., Sweet, M.J., Jennings, M.P., and Mcewan, A.G. (2010). The multi-copper-ion oxidase CueO of *Salmonella enterica* serovar Typhimurium is required for systemic virulence. *Infect Immun*. 78**,** 2312-2319.

Ammendola, S., Pasquali, P., Pistoia, C., Petrucci, P., Petrarca, P., Rotilio, G., and Battistoni, A. (2007). High-affinity Zn2+ uptake system ZnuABC is required for bacterial zinc homeostasis in intracellular environments and contributes to the virulence of *Salmonella enterica*. *Infect Immun*. 75**,** 5867-5876.

Angerer, A., Klupp, B., and Braun, V. (1992). Iron transport systems of *Serratia marcescens*. *J Bacteriol*. 174**,** 1378-1387.

Appia-Ayme, C., Hall, A., Patrick, E., Rajadurai, S., Clarke, T.A., and Rowley, G. (2012). ZraP is a periplasmic molecular chaperone and a repressor of the zinc-responsive two-component regulator ZraSR. *Biochem J*. 442**,** 85-93.

Bachman, M.A., Lenio, S., Schmidt, L., Oyler, J.E., and Weiser, J.N. (2012). Interaction of lipocalin 2, transferrin, and siderophores determines the replicative niche of *Klebsiella pneumoniae* during pneumonia. *MBio*. 3.

Bearden, S.W., Fetherston, J.D., and Perry, R.D. (1997). Genetic organization of the yersiniabactin biosynthetic region and construction of avirulent mutants in *Yersinia pestis*. *Infect Immun*. 65**,** 1659-1668.

Bearden, S.W., and Perry, R.D. (1999). The Yfe system of *Yersinia pestis* transports iron and manganese and is required for full virulence of plague. *Mol Microbiol*. 32**,** 403-414.

Benevides-Matos, N., and Biville, F. (2010). The Hem and Has haem uptake systems in *Serratia marcescens*. *Microbiology*. 156**,** 1749-1757.

Boyer, E., Bergevin, I., Malo, D., Gros, P., and Cellier, M.F. (2002). Acquisition of Mn(II) in addition to Fe(II) is required for full virulence of *Salmonella enterica* serovar Typhimurium. *Infect Immun*. 70**,** 6032-6042.

Braun, M., Endriss, F., Killmann, H., and Braun, V. (2003). *In vivo* reconstitution of the FhuA transport protein of *Escherichia coli* K-12. *J Bacteriol*. 185**,** 5508-5518.

Brown, N.L., Barrett, S.R., Camakaris, J., Lee, B.T., and Rouch, D.A. (1995). Molecular genetics and transport analysis of the copper-resistance determinant (pco) from *Escherichia coli* plasmid pRJ1004. *Mol Microbiol*. 17**,** 1153-1166.

Cao, J., Woodhall, M.R., Alvarez, J., Cartron, M.L., and Andrews, S.C. (2007). EfeUOB (YcdNOB) is a tripartite, acid-induced and CpxAR-regulated, low-pH Fe2+ transporter that is cryptic in *Escherichia coli* K-12 but functional in *E. coli* O157:H7. *Mol Microbiol*. 65**,** 857-875.

Chakraborty, R., Lemke, E.A., Cao, Z., Klebba, P.E., and Van Der Helm, D. (2003). Identification and mutational studies of conserved amino acids in the outer membrane receptor protein, FepA, which affect transport but not binding of ferric-enterobactin in *Escherichia coli*. *Biometals*. 16**,** 507-518.

Cox, G.B., Gibson, F., Luke, R.K., Newton, N.A., O'brien, I.G., and Rosenberg, H. (1970). Mutations affecting iron transport in *Escherichia coli*. *J Bacteriol*. 104**,** 219-226.

Crouch, M.L., Castor, M., Karlinsey, J.E., Kalhorn, T., and Fang, F.C. (2008). Biosynthesis and IroC-dependent export of the siderophore salmochelin are essential for virulence of *Salmonella enterica* serovar Typhimurium. *Mol Microbiol*. 67**,** 971-983.

Deiss, K., Hantke, K., and Winkelmann, G. (1998). Molecular recognition of siderophores: a study with cloned ferrioxamine receptors (FoxA) from *Erwinia herbicola* and *Yersinia enterocolitica*. *Biometals*. 11**,** 131-137.

Desrosiers, D.C., Bearden, S.W., Mier, I., Jr., Abney, J., Paulley, J.T., Fetherston, J.D., Salazar, J.C., Radolf, J.D., and Perry, R.D. (2010). Znu is the predominant zinc importer in *Yersinia pestis* during *in vitro* growth but is not essential for virulence. *Infect Immun*. 78**,** 5163-5177.

Dozois, C.M., Daigle, F., and Curtiss, R., 3rd (2003). Identification of pathogen-specific and conserved genes expressed in vivo by an avian pathogenic *Escherichia coli* strain. *Proc Natl Acad Sci U S A*. 100**,** 247-252.

Espariz, M., Checa, S.K., Audero, M.E., Pontel, L.B., and Soncini, F.C. (2007). Dissecting the *Salmonella* response to copper. *Microbiology*. 153**,** 2989-2997.

Fetherston, J.D., Mier, I., Jr., Truszczynska, H., and Perry, R.D. (2012). The Yfe and Feo transporters are involved in microaerobic growth and virulence of *Yersinia pestis* in bubonic plague. *Infect Immun*. 80**,** 3880-3891.

Forman, S., Paulley, J.T., Fetherston, J.D., Cheng, Y.Q., and Perry, R.D. (2010). Yersinia ironomics: comparison of iron transporters among *Yersinia pestis* biotypes and its nearest neighbor, *Yersinia pseudotuberculosis*. *Biometals*. 23**,** 275-294.

Gabbianelli, R., Scotti, R., Ammendola, S., Petrarca, P., Nicolini, L., and Battistoni, A. (2011). Role of ZnuABC and ZinT in *Escherichia coli* O157:H7 zinc acquisition and interaction with epithelial cells. *BMC Microbiol*. 11**,** 36.

Garcia, E.C., Brumbaugh, A.R., and Mobley, H.L. (2011). Redundancy and specificity of *Escherichia coli* iron acquisition systems during urinary tract infection. *Infect Immun*. 79**,** 1225-1235.

Ghigo, J.M., Letoffe, S., and Wandersman, C. (1997). A new type of hemophore-dependent heme acquisition system of *Serratia marcescens* reconstituted in *Escherichia coli*. *J Bacteriol*. 179**,** 3572-3579.

Grass, G., Fan, B., Rosen, B.P., Franke, S., Nies, D.H., and Rensing, C. (2001). ZitB (YbgR), a member of the cation diffusion facilitator family, is an additional zinc transporter in *Escherichia coli*. *J Bacteriol*. 183**,** 4664-4667.

Grass, G., Wong, M.D., Rosen, B.P., Smith, R.L., and Rensing, C. (2002). ZupT is a Zn(II) uptake system in *Escherichia coli*. *J Bacteriol*. 184**,** 864-866.

Grim, C.J., Kothary, M.H., Gopinath, G., Jarvis, K.G., Beaubrun, J.J., Mcclelland, M., Tall, B.D., and Franco, A.A. (2012). Identification and characterization of *Cronobacter* iron acquisition systems. *Appl Environ Microbiol*. 78**,** 6035-6050.

Hagan, E.C., and Mobley, H.L. (2009). Haem acquisition is facilitated by a novel receptor Hma and required by uropathogenic *Escherichia coli* for kidney infection. *Mol Microbiol*. 71**,** 79-91.

Hantke, K. (1990). Dihydroxybenzoylserine--a siderophore for *E. coli*. *FEMS Microbiol Lett*. 55**,** 5-8.

Hantke, K., Nicholson, G., Rabsch, W., and Winkelmann, G. (2003). Salmochelins, siderophores of *Salmonella enterica* and uropathogenic *Escherichia coli* strains, are recognized by the outer membrane receptor IroN. *Proc Natl Acad Sci U S A*. 100**,** 3677-3682.

Himpsl, S.D., Pearson, M.M., Arewang, C.J., Nusca, T.D., Sherman, D.H., and Mobley, H.L. (2010). Proteobactin and a yersiniabactin-related siderophore mediate iron acquisition in *Proteus mirabilis*. *Mol Microbiol*. 78**,** 138-157.

Hsieh, P.F., Lin, T.L., Lee, C.Z., Tsai, S.F., and Wang, J.T. (2008). Serum-induced iron-acquisition systems and TonB contribute to virulence in *Klebsiella pneumoniae* causing primary pyogenic liver abscess. *J Infect Dis*. 197**,** 1717-1727.

Hung, K.W., Tsai, J.Y., Juan, T.H., Hsu, Y.L., Hsiao, C.D., and Huang, T.H. (2012). Crystal structure of the *Klebsiella pneumoniae* NFeoB/FeoC complex and roles of FeoC in regulation of Fe2+ transport by the bacterial Feo system. *J Bacteriol*. 194**,** 6518-6526.

Izumiya, H., Sekizuka, T., Nakaya, H., Taguchi, M., Oguchi, A., Ichikawa, N., Nishiko, R., Yamazaki, S., Fujita, N., Watanabe, H., Ohnishi, M., and Kuroda, M. (2011). Whole-genome analysis of *Salmonella enterica* serovar Typhimurium T000240 reveals the acquisition of a genomic island involved in multidrug resistance via IS1 derivatives on the chromosome. *Antimicrob Agents Chemother*. 55**,** 623-630.

Johnson, T.J., Siek, K.E., Johnson, S.J., and Nolan, L.K. (2006). DNA sequence of a ColV plasmid and prevalence of selected plasmid-encoded virulence genes among avian *Escherichia coli* strains. *J Bacteriol*. 188**,** 745-758.

Kammler, M., Schon, C., and Hantke, K. (1993). Characterization of the ferrous iron uptake system of *Escherichia coli*. *J Bacteriol*. 175**,** 6212-6219.

Kehres, D.G., Janakiraman, A., Slauch, J.M., and Maguire, M.E. (2002). SitABCD is the alkaline Mn(2+) transporter of *Salmonella enterica* serovar Typhimurium. *J Bacteriol*. 184**,** 3159-3166.

Kehres, D.G., Zaharik, M.L., Finlay, B.B., and Maguire, M.E. (2000). The NRAMP proteins of *Salmonella* typhimurium and *Escherichia coli* are selective manganese transporters involved in the response to reactive oxygen. *Mol Microbiol*. 36**,** 1085-1100.

Kershaw, C.J., Brown, N.L., and Hobman, J.L. (2007). Zinc dependence of *zinT* (*yodA*) mutants and binding of zinc, cadmium and mercury by ZinT. *Biochem Biophys Res Commun*. 364**,** 66-71.

Kirillina, O., Bobrov, A.G., Fetherston, J.D., and Perry, R.D. (2006). Hierarchy of iron uptake systems: Yfu and Yiu are functional in *Yersinia pestis*. *Infect Immun*. 74**,** 6171-6178.

Koch, D., Chan, A.C., Murphy, M.E., Lilie, H., Grass, G., and Nies, D.H. (2011). Characterization of a dipartite iron uptake system from uropathogenic *Escherichia coli* strain F11. *J Biol Chem*. 286**,** 25317-25330.

Letoffe, S., Heuck, G., Delepelaire, P., Lange, N., and Wandersman, C. (2009). Bacteria capture iron from heme by keeping tetrapyrrol skeleton intact. *Proc Natl Acad Sci U S A*. 106**,** 11719-11724.

Leveille, S., Caza, M., Johnson, J.R., Clabots, C., Sabri, M., and Dozois, C.M. (2006). Iha from an *Escherichia coli* urinary tract infection outbreak clonal group A strain is expressed *in vivo* in the mouse urinary tract and functions as a catecholate siderophore receptor. *Infect Immun*. 74**,** 3427-3436.

Li, Y., Yin, J., Qu, G., Lv, L., Li, Y., Yang, S., and Wang, X.G. (2008). Gene cloning, protein purification, and enzymatic properties of multicopper oxidase, from *Klebsiella* sp. 601. *Can J Microbiol*. 54**,** 725-733.

Luck, S.N., Turner, S.A., Rajakumar, K., Sakellaris, H., and Adler, B. (2001). Ferric dicitrate transport system (Fec) of *Shigella flexneri* 2a YSH6000 is encoded on a novel pathogenicity island carrying multiple antibiotic resistance genes. *Infect Immun*. 69**,** 6012-6021.

Ma, L.C., Fang, C.T., Lee, C.Z., Shun, C.T., and Wang, J.T. (2005). Genomic heterogeneity in *Klebsiella pneumoniae* strains is associated with primary pyogenic liver abscess and metastatic infection. *J Infect Dis*. 192**,** 117-128.

Mahren, S., Schnell, H., and Braun, V. (2005). Occurrence and regulation of the ferric citrate transport system in *Escherichia coli* B, *Klebsiella pneumoniae*, *Enterobacter aerogenes*, and *Photorhabdus luminescens*. *Arch Microbiol*. 184**,** 175-186.

Makui, H., Roig, E., Cole, S.T., Helmann, J.D., Gros, P., and Cellier, M.F. (2000). Identification of the *Escherichia coli* K-12 Nramp orthologue (MntH) as a selective divalent metal ion transporter. *Mol Microbiol*. 35**,** 1065-1078.

Marolda, C.L., Valvano, M.A., Lawlor, K.M., Payne, S.M., and Crosa, J.H. (1987). Flanking and internal regions of chromosomal genes mediating aerobactin iron uptake systems in enteroinvasive *Escherichia coli* and *Shigella flexneri*. *J Gen Microbiol*. 133**,** 2269-2278.

Mermod, M., Magnani, D., Solioz, M., and Stoyanov, J.V. (2012). The copper-inducible ComR (YcfQ) repressor regulates expression of ComC (YcfR), which affects copper permeability of the outer membrane of *Escherichia coli*. *Biometals*. 25**,** 33-43.

Nielubowicz, G.R., Smith, S.N., and Mobley, H.L. (2010). Zinc uptake contributes to motility and provides a competitive advantage to *Proteus mirabilis* during experimental urinary tract infection. *Infect Immun*. 78**,** 2823-2833.

Noll, M., Petrukhin, K., and Lutsenko, S. (1998). Identification of a novel transcription regulator from *Proteus mirabilis*, PMTR, revealed a possible role of YJAI protein in balancing zinc in *Escherichia coli*. *J Biol Chem*. 273**,** 21393-21401.

Oelschlaeger, T.A., Zhang, D., Schubert, S., Carniel, E., Rabsch, W., Karch, H., and Hacker, J. (2003). The high-pathogenicity island is absent in human pathogens of *Salmonella enterica* subspecies I but present in isolates of subspecies III and VI. *J Bacteriol*. 185**,** 1107-1111.

Osman, D., Waldron, K.J., Denton, H., Taylor, C.M., Grant, A.J., Mastroeni, P., Robinson, N.J., and Cavet, J.S. (2010). Copper homeostasis in *Salmonella* is atypical and copper-CueP is a major periplasmic metal complex. *J Biol Chem*. 285**,** 25259-25268.

Outten, F.W., Huffman, D.L., Hale, J.A., and O'halloran, T.V. (2001). The independent cue and cus systems confer copper tolerance during aerobic and anaerobic growth in *Escherichia coli*. *J Biol Chem*. 276**,** 30670-30677.

Patzer, S.I., and Hantke, K. (1998). The ZnuABC high-affinity zinc uptake system and its regulator Zur in *Escherichia coli*. *Mol Microbiol*. 28**,** 1199-1210.

Payne, S.M., Wyckoff, E.E., Murphy, E.R., Oglesby, A.G., Boulette, M.L., and Davies, N.M. (2006). Iron and pathogenesis of *Shigella*: iron acquisition in the intracellular environment. *Biometals*. 19**,** 173-180.

Perez, J.M., Pradenas, G.A., Navarro, C.A., Henriquez, D.R., Pichuantes, S.E., and Vasquez, C.C. (2006). *Geobacillus stearothermophilus* LV *cadA* gene mediates resistance to cadmium, lead and zinc in *zntA* mutants of *Salmonella enterica* serovar Typhimurium. *Biol Res*. 39**,** 661-668.

Perry, R.D., Craig, S.K., Abney, J., Bobrov, A.G., Kirillina, O., Mier, I., Jr., Truszczynska, H., and Fetherston, J.D. (2012). Manganese transporters Yfe and MntH are Fur-regulated and important for the virulence of *Yersinia pestis*. *Microbiology*. 158**,** 804-815.

Perry, R.D., and San Clemente, C.L. (1979). Siderophore synthesis in *Klebsiella pneumoniae* and *Shigella sonnei* during iron deficiency. *J Bacteriol*. 140**,** 1129-1132.

Petrarca, P., Ammendola, S., Pasquali, P., and Battistoni, A. (2010). The Zur-regulated ZinT protein is an auxiliary component of the high-affinity ZnuABC zinc transporter that facilitates metal recruitment during severe zinc shortage. *J Bacteriol*. 192**,** 1553-1564.

Podladchikova, O., Rykova, V., Antonenka, U., and Rakin, A. (2012). *Yersinia pestis* autoagglutination is mediated by HCP-like protein and siderophore Yersiniachelin (Ych). *Adv Exp Med Biol*. 954**,** 289-292.

Pollack, J.R., Ames, B.N., and Neilands, J.B. (1970). Iron transport in *Salmonella* Typhimurium: mutants blocked in the biosynthesis of enterobactin. *J Bacteriol*. 104**,** 635-639.

Rakin, A., Schneider, L., and Podladchikova, O. (2012). Hunger for iron: the alternative siderophore iron scavenging systems in highly virulent *Yersinia*. *Front Cell Infect Microbiol*. 2**,** 151.

Rensing, C., Fan, B., Sharma, R., Mitra, B., and Rosen, B.P. (2000). CopA: An *Escherichia coli* Cu(I)-translocating P-type ATPase. *Proc Natl Acad Sci U S A*. 97**,** 652-656.

Rensing, C., Mitra, B., and Rosen, B.P. (1997). The *zntA* gene of *Escherichia coli* encodes a Zn(II)-translocating P-type ATPase. *Proc Natl Acad Sci U S A*. 94**,** 14326-14331.

Rensing, C., Mitra, B., and Rosen, B.P. (1998). A Zn(II)-translocating P-type ATPase from *Proteus mirabilis*. *Biochem Cell Biol*. 76**,** 787-790.

Rossi, M.S., Fetherston, J.D., Letoffe, S., Carniel, E., Perry, R.D., and Ghigo, J.M. (2001). Identification and characterization of the hemophore-dependent heme acquisition system of *Yersinia pestis*. *Infect Immun*. 69**,** 6707-6717.

Runyen-Janecky, L., Dazenski, E., Hawkins, S., and Warner, L. (2006). Role and regulation of the *Shigella flexneri* Sit and MntH systems. *Infect Immun*. 74**,** 4666-4672.

Runyen-Janecky, L.J., Reeves, S.A., Gonzales, E.G., and Payne, S.M. (2003). Contribution of the *Shigella flexneri* Sit, Iuc, and Feo iron acquisition systems to iron acquisition *in vitro* and in cultured cells. *Infect Immun*. 71**,** 1919-1928.

Russo, T.A., Carlino, U.B., and Johnson, J.R. (2001). Identification of a new iron-regulated virulence gene, *ireA,* in an extraintestinal pathogenic isolate of *Escherichia coli*. *Infect Immun*. 69**,** 6209-6216.

Sabri, M., Caza, M., Proulx, J., Lymberopoulos, M.H., Bree, A., Moulin-Schouleur, M., Curtiss, R., 3rd, and Dozois, C.M. (2008). Contribution of the SitABCD, MntH, and FeoB metal transporters to the virulence of avian pathogenic *Escherichia coli* O78 strain chi7122. *Infect Immun*. 76**,** 601-611.

Sabri, M., Houle, S., and Dozois, C.M. (2009). Roles of the extraintestinal pathogenic *Escherichia coli* ZnuACB and ZupT zinc transporters during urinary tract infection. *Infect Immun*. 77**,** 1155-1164.

Sabri, M., Leveille, S., and Dozois, C.M. (2006). A SitABCD homologue from an avian pathogenic *Escherichia coli* strain mediates transport of iron and manganese and resistance to hydrogen peroxide. *Microbiology*. 152**,** 745-758.

Sauer, M., Hantke, K., and Braun, V. (1990). Sequence of the *fhuE* outer-membrane receptor gene of *Escherichia coli* K12 and properties of mutants. *Mol Microbiol*. 4**,** 427-437.

Schubert, S., Cuenca, S., Fischer, D., and Heesemann, J. (2000). High-pathogenicity island of *Yersinia pestis* in enterobacteriaceae isolated from blood cultures and urine samples: prevalence and functional expression. *J Infect Dis*. 182**,** 1268-1271.

Stojiljkovic, I., and Hantke, K. (1992). Hemin uptake system of *Yersinia enterocolitica*: similarities with other TonB-dependent systems in gram-negative bacteria. *EMBO J*. 11**,** 4359-4367.

Thompson, J.M., Jones, H.A., and Perry, R.D. (1999). Molecular characterization of the hemin uptake locus (*hmu*) from *Yersinia pestis* and analysis of *hmu* mutants for hemin and hemoprotein utilization. *Infect Immun*. 67**,** 3879-3892.

Torres, A.G., and Payne, S.M. (1997). Haem iron-transport system in enterohaemorrhagic *Escherichia coli* O157:H7. *Mol Microbiol*. 23**,** 825-833.

Tree, J.J., Kidd, S.P., Jennings, M.P., and Mcewan, A.G. (2005). Copper sensitivity of *cueO* mutants of *Escherichia coli* K-12 and the biochemical suppression of this phenotype. *Biochem Biophys Res Commun*. 328**,** 1205-1210.

Tsolis, R.M., Baumler, A.J., Heffron, F., and Stojiljkovic, I. (1996). Contribution of TonB- and Feo-mediated iron uptake to growth of *Salmonella* Typhimurium in the mouse. *Infect Immun*. 64**,** 4549-4556.

Wang, D., and Fierke, C.A. (2013). The BaeSR regulon is involved in defense against zinc toxicity in *E. coli*. *Metallomics*. 5**,** 372-383.

Warner, P.J., Williams, P.H., Bindereif, A., and Neilands, J.B. (1981). ColV plasmid-specific aerobactin synthesis by invasive strains of *Escherichia coli*. *Infect Immun*. 33**,** 540-545.

Waters, L.S., Sandoval, M., and Storz, G. (2011). The *Escherichia coli* MntR miniregulon includes genes encoding a small protein and an efflux pump required for manganese homeostasis. *J Bacteriol*. 193**,** 5887-5897.

Wei, Y., and Fu, D. (2006). Binding and transport of metal ions at the dimer interface of the *Escherichia coli* metal transporter YiiP. *J Biol Chem*. 281**,** 23492-23502.

Williamson, N.R., Simonsen, H.T., Harris, A.K., Leeper, F.J., and Salmond, G.P. (2006). Disruption of the copper efflux pump (CopA) of *Serratia marcescens* ATCC 274 pleiotropically affects copper sensitivity and production of the tripyrrole secondary metabolite, prodigiosin. *J Ind Microbiol Biotechnol*. 33**,** 151-158.

Wyckoff, E.E., Lopreato, G.F., Tipton, K.A., and Payne, S.M. (2005). *Shigella dysenteriae* ShuS promotes utilization of heme as an iron source and protects against heme toxicity. *J Bacteriol*. 187**,** 5658-5664.

Zulfiqar, S., and Shakoori, A.R. (2012). Molecular characterization, metal uptake and copper induced transcriptional activation of efflux determinants in copper resistant isolates of *Klebsiella pneumoniae*. *Gene*. 510**,** 32-38.
